# Multicomponent Synthesis of C(8)‐Substituted Purine Building Blocks of Peptide Nucleic Acids from Prebiotic Compounds

**DOI:** 10.1002/open.202400265

**Published:** 2024-10-17

**Authors:** Eleonora Mancin, Eliana Capecchi, Lorenzo Botta, Bruno Mattia Bizzarri

**Affiliations:** ^1^ Department of Biological and Ecological Sciences University of Tuscia Viterbo 01100 Italy

**Keywords:** Multicomponent Synthesis, Peptide Nucleic Acid, C(8)-substituted purines, PNA's building block, Prebiotic synthesis

## Abstract

We have explored the reaction of a three‐components mixture of aminomalononitrile, urea and α‐amino acid methyl esters for the multicomponent synthesis substituted purines resembling PNA's building blocks. 2,6‐diamino‐purines, 6‐amino‐3,9‐dihydro‐2H‐purin‐2‐one (iso‐guanines), and 3,9‐dihydro‐6H‐purin‐6‐one derivatives, selectively decorated at C(8)‐position of the purine ring with different amino acid residues, were obtained from acceptable to good yields. The regio‐selectivity of the transformation was controlled by the use of urea in the ternary mixture and by the annulation agent involved in the ring‐closure of amino‐imidazole carbonitrile intermediates. Solvent free conditions, microwave irradiation and simple one‐carbon containing reagents further satisfied the major requirement of atom economy and sustainable chemistry. Due to the prebiotic nature of the three‐components mixture and of annulation agents, it also embodies the possibility for the synthesis of novel PNAs bearing purine nucleobases decorated at C(8)‐position of the imidazole ring as alternative RNA analogues in molecular evolution.

## Graphical abstract

Prebiotic reagents (AMN, Amino‐acids, Urea, Guanidine and Formic Acid) react via moder or ancestral multicomponent chemistry to produce selected C8‐PNA's derivatives. The synthesis is effective under MW assistance in MeCN and H_2_O as solvents.

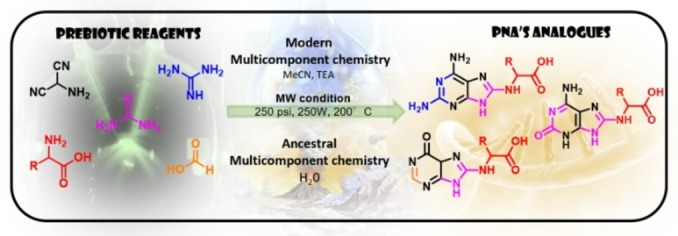

## Introduction

**Scheme 1:**
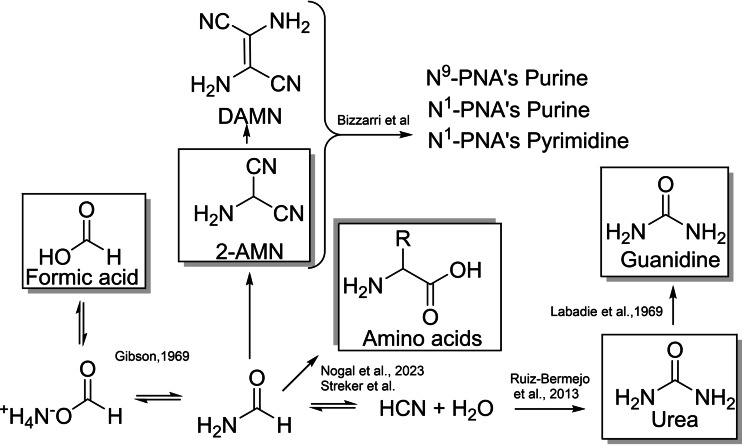
Chemistry of PNA's derivatives and related condensing agents.

The pioneering Miller‐Urey experiment highlighted possible pathways to set molecular evolution, which involve electric discharge and plasma and primitive atmosphere combined together to improve chemical complexity.^[1]^ In the modifications of the original experimental set, amino acids and hydroxy acids were synthesized and identified as chemical precursors for the pre‐metabolic machinery. Successively, RNA's nucleobases were obtained from a variety of gas mixtures and energy sources, and the unprecedented catalytic role of minerals and metal oxides in these transformations was analyzed in detail.[^2^, ^3^, ^4^, ^5^, ^6^] Building blocks of peptide nucleic acids (PNAs)^[7]^ were also isolated besides to canonical nucleobases, suggesting that PNAs, and PNAs like molecules, may have co‐evolved with RNA.[^8^, ^9^] In this latter case, the structural integrity of PNAs is favored by the 2‐aminoethylglycine motif instead of labile sugar–phosphate backbone, the nucleobase being linked to pseudopeptide by carboxy‐methylene spacer.^[10]^ The exploration of the chemical space of nucleobases by decoration with amino acid opens new possibility of alternative PNA's structures.[^11^, ^12^] We recently reported that aminomalononitrile (AMN) and diaminomaleonitrile (DAMN) are effective precursors for the synthesis of PNA's building blocks decorated at N(9), and N(1)‐positions of the purine ring, as well as at N(1)‐position of the pyrimidine ring.[^13^, ^14^, ^15^] The reaction proceeds by formation of imidazole intermediates, followed by heterocyclic annulation with simple compounds of prebiotic relevance, such as formic acid, urea and guanidine. In these experimental conditions, the scaffold morphing of the reaction (that is the formation of purine versus pyrimidine derivatives) was controlled by the appropriate energy source (thermal and photothermal conditions), favoring different reaction pathways. Prebiotic relationships between AMN, DAMN and annulation agents, and the reaction pathways to different PNA's building blocks, are reported in Scheme 1.[^13^, ^15^, ^16^, ^17^, ^18^, ^19^, ^20^, ^21^] Interestingly, novel PNA's derivatives showed high biological activity and good selectivity against a broad spectrum of RNA, DNA and retro‐viruses.[^13^, ^15^, ^22^]

Purine derivatives decorated at C(8)‐position of the heterocyclic ring may be of particular interest for the synthesis of biologically active PNAs. C(8)‐oxo‐purines are involved in non‐enzymatic primer extension,^[23]^ and they are template in the assembly of complex networks of nucleotides.^[24]^ In addition, 8‐thio‐adenine is a key intermediate in the prebiotic synthesis of 2′‐deoxyriboadenosine,[^25^, ^26^, ^27^] and C(8)‐substituted purines are inhibitors of respiratory syncytial virus^[28]^ and herpes simplex virus (type 1),^[29]^ showing high affinity towards human adenosine A1 receptor.^[30]^ The synthesis of C(8)‐substituted purines usually requires transition‐metal catalysis and cross‐coupling reactions encompassing toxic reagents, such as organotin,[^31^, ^32^, ^33^, ^34^] organozinc,^[35]^ alkylaluminium,[^36^, ^37^] alkylboronic acids,^[38]^ and Grignard reagents.[^39^, ^40^, ^41^] These procedures are not sustainable, and require expensive ligands, pre‐activation of the reagents, and anhydrous and/or anaerobic conditions.^[42]^ In order to overcome these drawbacks, and with the aim to design a sustainable synthesis of alternative PNA's purine nucleobases, we describe here the synthesis of C(8)‐substituted purine derivatives decorated with α‐amino‐acid residues, taking inspiration from previous prebiotic chemistry studies. The reaction involves the multicomponent synthesis of C(2)‐substituted amino imidazole carbonitriles from the ternary mixture of AMN, urea and α‐amino‐acid esters in the presence of triethylamine (TEA), followed by domino ring annulation with formic acid, urea and guanidine. The use of eco‐friendly solvents and microwave irradiation conditions improved the sustainability of the overall transformation. Finally, we moved toward original prebiotic multicomponent chemistry, overcoming the use of TEA and of organic solvents, and using α‐amino‐acids instead of the corresponding methyl ester derivatives.

## Results and Discussion

### Synthesis of C(2)‐Substituted Ammino‐Imidazole Carbonitrile Derivatives 4 a–d

**Scheme 2:**
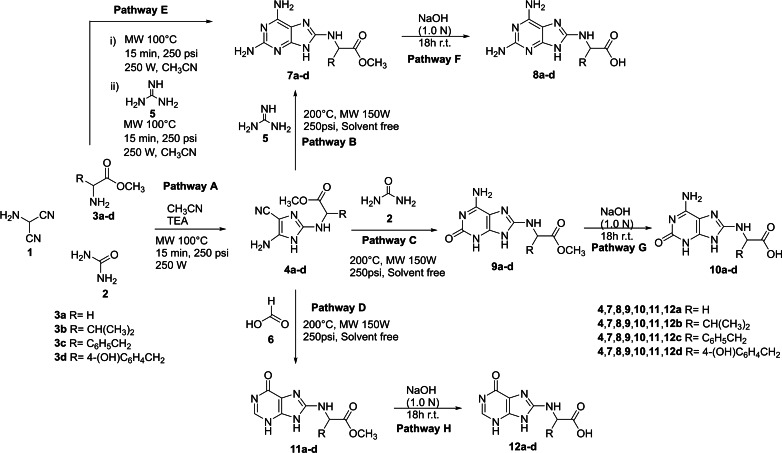
Multicomponent synthesis of C(8)‐substituted purines from the ternary mixture of AMN **1**, urea **2**, and α‐amino acid methyl esters **3 a**–**d**. Pathway A: preparation of amino imidazole carbonitriles **4 a**–**d**. Pathway B: preparation of C(8)‐substituted 2,6‐diamino‐purine methyl esters **7 a**–**d**. Pathway C: preparation of C(8)‐substituted iso‐guanine methyl esters **9 a**–**d**. Pathway D: preparation of C(8)‐substituted 3,9‐dihydro‐6H‐purin‐6‐one methyl esters **11 a**–**d**. Pathway E: one‐pot preparation of C(8)‐substituted 2,6‐diamino‐purine methyl ester **7 a**. Pathways F−H: preparation of C(8)‐substituted purine carboxylic acids **8 a**–**d, 10 a**–**d** and **12 a**–**d**.

C(2)‐substituted amino imidazole carbonitriles **4 a**–**d** were synthesized by a multicomponent procedure involving the condensation of AMN **1**, urea **2**, and α‐amino acid methyl esters **3 a**–**d** (glycine **3 a**, valine **3 b**, phenylalanine **3 c**, and tyrosine **3 d**), in the presence of TEA (Scheme 2, Pathway A).

**Scheme 3:**
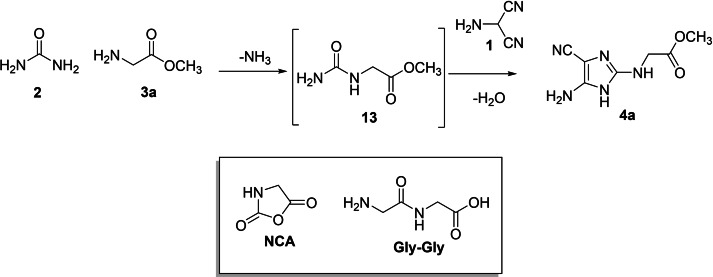
Proposed reaction pathway for the synthesis of **4 a**.

Reaction conditions were investigated for the synthesis of **4 a** from glycine **3 a** as a selected case. Different molar ratio of compounds **1**, **2**, and **3 a** (from 1 : 1.2 : 1.4 to 1 : 1.4 : 1.6), reaction solvents (solvent free condition, THF, 2‐MeTHF and CH_3_CN), temperature (from 25 °C to 250 °C), and energy sources (microwave and thermal heating) were studied. Results are reported in Table 1 (entries 1–9). The optimal experimental conditions were consistent with 1 : 1.4 : 1.2 molar ratio of the ternary mixture, TEA (1.2 eq), and MW irradiation (250 W, 250 psi) in CH_3_CN (3.0 mL) at 200 °C for 15 min (Table 1; entry 9). CH_3_CN is a well‐recognized prebiotic compound,^[43]^ with high solvent capacity.^[44]^ MW irradiation were generally more effective than heat (Table 1; entries 6,7,9 vs entries 1–5,8), minimizing the formation of polar AMN oligomers as side‐products, the increasing of temperature above 200 °C (220 °C or 250 °C) determined a significative decreasing of the yields (45 % vs 35 % vs 20 %).⋅ Compound **4 a** was probably obtained by initial condensation of **2** with **3 a** to yield *N*‐carbamoyl glycine methyl ester **13**, followed by reaction of **13** with **1** (Scheme 3).[^4^, ^45^] In accordance with this hypothesis, *N*‐carbamoyl glycine methyl ester **13** was isolated in the reaction mixture. Traces amount of Gly–Gly and *N*‐carboxy anhydride (NCA) were also detected, the latter compound being a well‐recognized condensing agent in prebiotic processes involving amino acids.^[46]^

**Table 1:** Amino imidazole carbonitrile methyl esters **4 a**–**d** from three component condensation of AMN, urea and α‐amino acids.

Entry	α‐Amino acid residues	Conditions	Product	R	Yield (%)^[d]^
1	Glycine	r. t. 6 day, THF^[a]^	**4 a**	H	6
2		100 °C 24 h, THF^[a]^			–
3		100 °C 24 h, THF^[b]^			3
4		100 °C 24 h, THF^[c]^			5
5		100 °C 24 h, 2‐Me‐THF^[a]^			6
6		M.W. 250 psi, 250 W, 150 °C 15 min, THF^[a]^			6
7		M.W. 250 psi, 250 W, 200 °C 15 min, solvent free^[a]^			7
8		100 °C 24 h, CH_3_CN^[a]^			17
9		M.W. 250 psi, 250 W, 200 °C 15 min, CH_3_CN^[a]^			45, 35^[e]^, 20^[f]^
10	Valine		**4 b**	CH(CH_3_)_2_	39
11	Phenylalanine		**4 c**	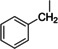	65
12	Tyrosine		**4 d**	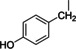	53

[a] **1** (0,20 mmol), **2** (0,28 mmol), triethylamine (0,24 mmol) and **3 a**–**d** (0,24 mmol). [b] **1** (0,20 mmol), **2** (0,28 mmol), triethylamine (0,24 mmol) and **3 a**–**d** (0,28 mmol). [c] **1** (0,20 mmol), **2** (0,32 mmol), triethylamine (0,24 mmol) and **3 a**–**d** (0,24 mmol). [d] Yield is referred to the conversion of **1**. [e] **1** (0,20 mmol), **2** (0,28 mmol), triethylamine (0,24 mmol) and **3 a** (0,24 mmol), M.W. 250 psi, 250 W, 220 °C 15 min, CH_3_CN. [f] **1** (0,20 mmol), **2** (0,28 mmol), triethylamine (0,24 mmol) and **3 a** (0,24 mmol), M.W. 250 psi, 250 W, 220 °C 15 min, CH_3_CN.

**Scheme 4:**
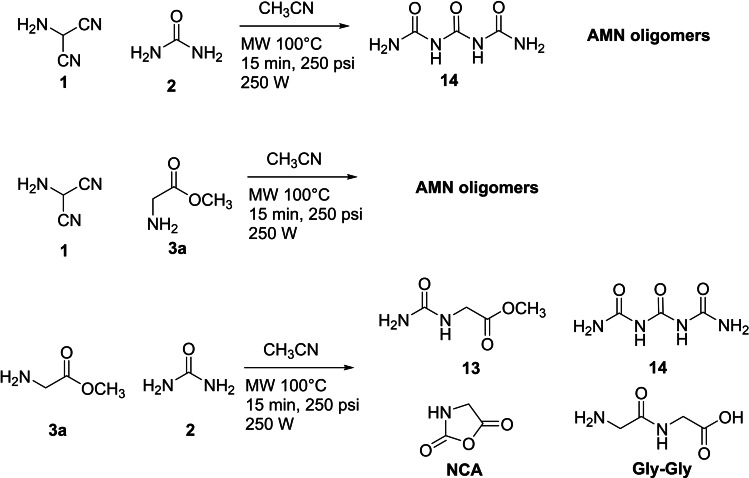
Two component reactions designed on the basis of different combination of compounds **1**, **2** and **3 a**.

A set of different combination of the three reagents was performed to further confirm the suggested reaction pathway (Scheme 4). The condensation of **1** with **2** afforded triuret **14** as the main reaction product besides AMN oligomers (SI#2),[^14^, ^47^, ^48^, ^49^, ^50^] and AMN oligomers were the only products obtained from the reaction of **1** with **3 a**.^[6]^ As expected, the reaction of **2** with **3 a** afforded **13** in appreciable yield (30 %), besides to **14** (12 %), NCA (14 %) and Gly–Gly (5 %), confirming the pivotal role of urea in the control of regioselectivity of the first step of the transformation (reaction pathways for the formation of **14**, NCA and Gly–Gly are in SI#2, Scheme S3).

The synthetic scope of the reaction was generalized to α‐ammino‐acid methyl esters **3 b‐d** bearing aliphatic and aromatic side‐chains. Amino imidazole carbonitriles **4 b**–**d** were obtained from acceptable to good yields (Scheme 2; Table 1, entries 10–11). Structural data of compounds **4 b**–**d** are in SI#1. The presence of aromatic substituents improved the overall yield of the reaction (Table 1, entries 11–12). Examples of the reactivity of α‐amino acids in multicomponent chemistry are reported, and the role played by the isoelectric point, as well as by hydrogen bonding and n→π interactions between adjacent amino acids, discussed in detail.^[49]^ A similar trend of reactivity was observed in the synthesis of 1H‐purin‐6‐one derivatives,^[13]^ amino‐imidazoles,^[14]^ *N*‐9 di‐aminopurines and guanine analogues.^[15]^ In these latter cases, the pKa and nucleophilicity of aromatic amino acids were critical parameters for the efficacy of the reaction.

### Synthesis of C(8)‐Substituted Purines

Compounds **4 a**–**d** were used as starting material for the synthesis of C(8)‐substituted 2,6‐diamino‐purines **7 a**–**d** and **8 a**–**d**, 6‐amino‐3,9‐dihydro‐2H‐purin‐2‐one derivatives (iso‐guanines) **9 a**–**d** and **10 a**–**d**, and 3,9‐dihydro‐6H‐purin‐6‐one derivatives **11 a**–**d** and **12 a**–**d**, by applying annulation protocols with formic acid, urea and guanidine (Scheme 2).[^13^, ^51^] As a general procedure, compounds **4 a**–**d** (0,28 mmol) were reacted with the selected annulation agent, guanidine carbonate **5** (0,56 mmol) (Scheme 2, Pathway B), urea **2** (0,56 mmol) (Scheme 2, Pathway C), and formic acid **6** (0,56 mmol) (Scheme 2, Pathway D), in solvent free conditions^[52]^ under MW irradiation (2.0 min 200 °C, 150 W, 250 psi). Methyl ester derivatives **7 a**–**d**, **9 a**–**d**, and **11 a**–**d** were obtained from appreciable to high yield (Table 2) by simple work‐up of the reaction mixture with AcOEt and H_2_O (SI#1). The annulation process was more effective for aromatic **4 c** and **4 d** than other cases (Table 2, entries 7–12 vs entries 1–6). Urea showed a low annulation efficacy, probably due to the formation of the undesired biuret and oligomers (Scheme 4).[^47^, ^48^] Next, the one pot synthesis of C(8)‐substituted purines was evaluated without isolation of the amino imidazole carbonitrile intermediate, analyzing the case of **3 a** as a representative example. AMN **1** (1.0 eq), urea **2** (1.4 eq) and glycine methyl ester **3 a** (1.2 eq) were irradiated with MW (250 W) assistance at 200 °C for 2 min in CH_3_CN, and the crude added with **5** (2.0 eq) without any preliminary work‐up, followed by irradiation with MW (250 W) at 200 °C for 2 min. Under this experimental conditions **7 a** was isolated in a total yield comparable to that previously obtained by the domino (two steps) procedure (Scheme 2, Pathway E). C(8)‐substituted purine derivatives **7 a**–**d**, **9 a**–**d**, and **11 a**–**d** were converted into the corresponding carboxylic‐acids **8 a**–**d**, **10 a**–**d** and **12 a**–**d** in quantitative yields by standard hydrolysis conditions (NaOH 1.0 N) at 25 °C (Scheme 2, Pathways F–H) (SI#1). These latter compounds are suitably to be directly coupled with pseudopeptide backbone 2‐aminoethyl glycine in order to obtain novel PNAs.^[53]^

**Table 2:** Synthesis of diaminopurine analogues **7 a**‐**d**, aminopurine analogues **9 a‐d**, and isoguanine analogues **11 a**–**d**.

	α‐Amino acid	Condition^[a]^	Product	R	Yield (%)^[b]^
1	Glycine	Solvent free MW 2.0 min 200 °C, 150 W, 250 psi	**7 a**	H	51, 42^c^
2			**9 a**		26
3			**11 a**		39
4	Valine		**7 b**	CH(CH_3_)_2_	30
5			**9 b**		22
6			**11 b**		73
7	Phenylalanine		**7 c**	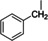	88
8			**9 c**		84
9			**11 c**		86
10	Tyrosine		**7 d**	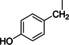	58
11			**9 d**		35
12			**11 d**		47

[a] compound **4 a**–**d** (0,28 mmol) and carbon donor source **2** or **5** or **6** (0,56 mmol) for 2 min at 200 °C in MW assistance. [b] Yield have been calculated with respect to converted C‐2 imidazole derivatives **4 a**–**d**. The conversion of the reaction was evaluated on the basis of unreacted **4 a**–**d**. [c] Yield is referred to the one pot two steps procedure.

**Scheme 5:**
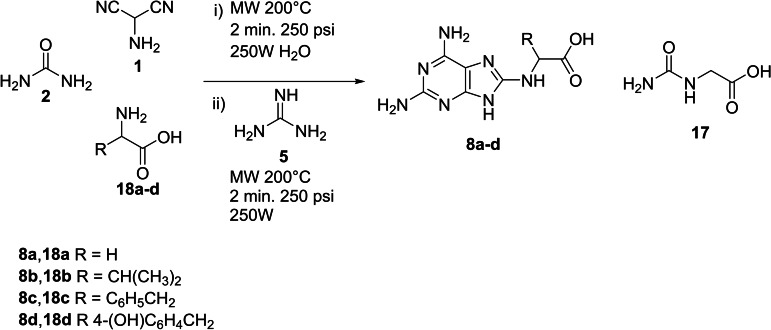
Preparation of C(8)‐substituted 2,6‐diamino‐purine acetic acids **8 a**–**d** in H_2_O, overcoming TEA and using α‐amino‐acids instead of the corresponding methyl esters.

Finally, the efficacy of the multicomponent reaction was studied under more reasonable prebiotic conditions in H_2_O, overcoming TEA and using α‐amino‐acids instead of the corresponding methyl esters. Briefly, AMN **1** (1.0 eq), urea **2** (1.4 eq), and α‐amino acids **13 a**–**d** (1.2 eq) were irradiated with MW (250 W) in H_2_O at 200 °C for 2 min (Scheme 5). Guanidine (2.0 eq) was added to the reaction mixture and the crude was irradiated with MW (250 W) at 200 °C for 2 min.

The yields of **8 a**–**d** were evaluated by HPLC by comparison with original samples after simple extraction of the reaction mixture with AcOEt and H_2_O mixture (1 : 1 v/v) (Table 3, entries 1–4). Compounds **8 a**–**d** were obtained in appreciable yield, besides hydantoic acid **17**, unreacted AMN and different unidentified products. These latter were expected as a result of the chemical complexity for unselective prebiotic processes (Table 3, entries 1–4). Again, aromatic amino acids afforded C(8)‐substituted purines in yield higher than aliphatic counterpart, confirming previous reactivity data (chromatographic profiles are in SI#3).

**Table 3:** Synthesis of C(8)‐substituted 2,6‐diamino‐purine acetic acids **8 a**–**d** under prebiotic conditions.

Entry	α‐Amino acid	Condition^[a]^	Products	Yield (%)^[b]^
1	Glycine	H_2_O, MW 200 °C, 250 W 250 psi 2 min.	**8 a**	8
2	Valine		**8 b**	5
3	Phenylalanine		**8 c**	15
4	Tyrosine		**8 d**	10

[a] AMN **1** (0,20 mmol), urea **2** (0,28 mmol), α‐amino acid **13 a**–**d** (0,24 mmol) were dissolved in CH_3_CN and under MW assistance at 200 °C for 2 min. Guanidine (0,40 mmol) was added and the reaction mixture was irradiated at 200 °C for 2 min. [b] Yield have been calculated with respect to converted AMN **1**. The conversion of the reaction was evaluated on the basis of unreacted **1**.

## Conclusions

The multicomponent synthesis of C(8)‐substituted purine derivatives decorated with different α‐amino‐acid residues has been developed taking inspiration from the chemistry of the origins. It involves prebiotic compounds, such as AMN, urea, α‐amino‐acids, guanidine, and formic acid. AMN is prebiotically obtained from universally ubiquitous HCN, amino acids are facile products of HCN chemistry, urea and guanidine are product of HCN condensation, and formic acid is easily produced from prebiotic formamide and ammonium formate.^[54]^ The use of water, eco‐friendly organic solvents, and microwave assisted irradiation, improved the atom economy and sustainability of the process. The reaction proceeds in two steps: i) the multicomponent synthesis of amino C(2)‐substituted imidazole carbonitrile intermediates from the ternary mixture of AMN, urea and α‐amino‐acids, in the presence of TEA; ii) microwave assisted annulation of C(2)‐substituted imidazole carbonitrile intermediates with urea, formic acid and guanidine. In the first step, the regioselectivity of the condensation was controlled by urea. We observed that the overall yield of the process was dependent by the α‐amino acid, the presence of aromatic substituents affording the best results. The synthetic procedure was effective when performed without TEA. The use of free amino acids and H_2_O activated different reaction pathways and self‐condensation processes providing expected high chemical complexity. The comparison between the two multicomponent approaches highlighted the higher selectivity and yield of the reaction with amino acid methyl esters than simple amino acids, CH_3_CN favoring the solubility of the reagents. On the other hand, the higher chemical variability observed with no protected amino acids in solvent free conditions provides, in principle, variety and structural complexity typical of Darwinian molecular evolution.[^48^, ^49^]

## Material and Methods

### Materials

All solvents and reagents were purchased from Aldrich Chemical Co. (purity grade >99 %). Monitoring and purification of the reactions have been performed with silica gel 60 and silica 60‐F254 acquired from Merck. Visualization of plates has been performed using a UV lamp at 254 nm. All products were completely dried under high vacuum (10–3 mbar) prior to the spectroscopic characterization. All of the NMR spectra were acquired on a Bruker Advance DRX400 (400 MHz/100 MHz) spectrometer. Signals and chemical shifts of the reported ^1^H and ^13^C‐NMR spectra are in parts per million and internally referenced to DMSO‐*d*6; CdCl_3_ and MeOD‐*d*4. Coupling constants (*J*) are reported in Hz. Multiplicities are reported as follows: s=singlet, d=doublet, t=triplet, dd=double doublets, m=multiplet. UHPLC were performed on Ultimate 3000 Rapid Resolution system (DIONEX, Sunnyvale, USA) using Hypersil GOLD™ (3 μm, 150 mm 4.6 mm) or by a Vanquish U‐HPLC (Thermo scientific) associated to ISQ‐EC MS(Thermo scientific) equipped with C18 Avantor ACE‐5 (5 μm×250 mm×4.6 mm).

### General procedure for the synthesis of imidazoles 4 a–d

To a solution of Urea **2** (0.28 mmol) in CH_3_CN (3 mL) was added triethylamine (0.24 mmol) and a selected alpha amino‐acid methyl ester **3 a**–**d** (0.24 mmol). The mixture was stirred at room temperature for 30 min. To this solution was added aminomalononitrile p‐toluenesulfonate **1** (0.20 mmol), and the solution was stirred under microwave condition using the following program:

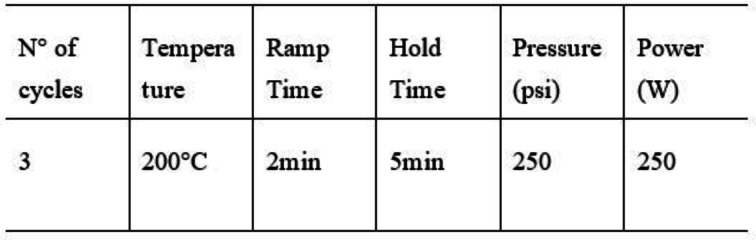




At the end of the reaction, the solvent was removed under reduced pressure and the residue was dissolved in ethyl acetate (10 mL) and extracted with H_2_O (3×10 mL) The organic layer was dried over Na_2_SO_4_, and the solvent was evaporated under reduced pressure. The crude was purified by flash‐chromatography eluting with dietil ether/hexane (6 : 1) to afford **4 a**–**d** from 25 to 45 % of yield.

### General Procedure for the Synthesis of Diaminopurine 7 a‐d, Isoguanine 9 a‐d and Aminopurine 11 a–d

Imidazole **4 a**–**d** (0.20 mmol) and guanidine carbonate **5** (for derivatives **7 a**–**d**) or urea **2** (for derivatives **9 a**–**d**) or formic acid **6** (for derivates **11 a**–**d**) (0,56 mmol) were irradiated under microwave conditions using the following program:

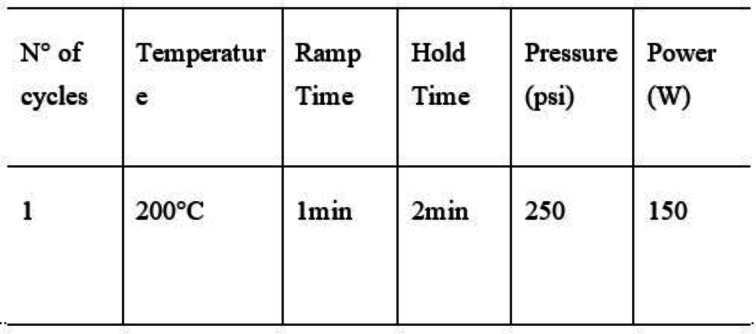




The solution stirred under microwave condition above described. At the end of the reaction, the mixture was dissolved in ethyl acetate (10 mL) and extracted with H_2_O (3×10 mL) The organic layer was dried over Na_2_SO_4_, and the solvent was evaporated under reduced pressure. Compounds **7 a**–**d**, **9 a**–**d** and **11 a**–**d** were obtained with yield from 12 to 88 %.

### General Procedure for the Synthesis of Diaminopurine 8 a–d by Prebiotic Chemistry Approach

To a solution of Urea **2** (0.28 mmol) in water (3 mL) was added triethylamine (0.24 mmol) and a selected alpha amino‐acid methyl estyer **3 a**–**d** (0.24 mmol). The mixture was stirred at room temperature for 30 min. To this solution was added aminomalononitrile p‐toluenesulfonate **1** (0.20 mmol), and the solution was stirred under microwave condition using the following program:

The solution stirred under microwave condition above described. At the end of the reaction the solvent was removed under reduced pressure and the residue was dissolved in ethyl acetate (10 mL) and extracted with H_2_O (3×10 mL) The organic layer was dried over Na_2_SO_4_, and the solvent was evaporated under reduced pressure. Then, guanidine **5** (0,56 mmol) was added to the crude of reaction and the mixture was stirred under microwave condition by the same condition previously describe. Finally, the crude was dissolved in ethyl acetate (10 mL) and extracted with H_2_O (3×10 mL) The organic layer was dried over Na_2_SO_4_, and the solvent was evaporated under reduced pressure. Compounds **8 a**–**d** were detected in the crude using the Ultimate 3000 Rapid Resolution UHPLC system (DIONEX, Sunnyvale, USA) equipped with C18 Hypersil GOLD™ column (3 μm×150 mm×4.6 mm). Chromatographic separations were achieved using the following conditions: column temperature 25 °C, flow rate 1.0 ml/min, gradient elution with phase A (H_2_O, 0.1 % formic acid) and phase B (acetonitrile, CH_3_CN). The gradient employed was as follows: 5 min 90 % A; 35 min 70 % A; 45 min 90 % A. Products were detected by their absorbance at 254 nm with yield from 5 to 15 %.

### General Procedure for the Synthesis of Diaminopurine, Isoguanine and Aminopurine Analogues Bearing Free Carboxylic Acid Moiety (7 a–d, 9 a–d and 11 a–d)

Compounds **7 a**–**d** or **9 a**–**d** or **11 a**–**d** (0.10 mmol) were treated with an aqueous solution of NaOH (1.0 N, 1.0 mL) and stirred for 18 h at room temperature. The solution was acidified with HCl 1.0 N until reaching neutral pH, freeze‐dried, and washed with methanol. Compound **8 a**–**d**, **10 a**–**d** and **12 a**–**d** were obtained in quantitative yield after evaporation of the solvent.

### Spectroscopic Data

*Compound 4a* Methyl (5‐amino‐4‐cyano‐1H‐imidazol‐2‐yl)glycinate.The crude residue was purified by silica gel chromatography eluting with Dietil ether/Hexane (6 : 1). ^1^H‐NMR (400 MHz, DMSO‐*d*6, ppm): δ 7.97 (s, 1H, NH), 6.80 (s, 1H, NH_2_), 6.24 (s, 1H, NH), 3.96‐3.88 (m, CH_2_), 3.64 (s, 3H, O−CH_3_), ^13^C‐NMR (100 MHz, DMSO‐*d*6, ppm): δ 170.76 (C=O), 157.09 (C), 139.09 (C), 116.75 (C), 89.41 (C), 55.29 (O−CH_3_), 31.13 (CH_2_). MS (ESI): *m*/*z* (M+H) +196.08. Elemental analysis for C_7_H_9_N_5_O_2_ calcd C, 43.08; H, 4.65; N, 35.88; O, 16.39. Found: C, 43.10; H, 4.67; N, 35.90; O, 16.35.

*Compound 4b* Methyl (5‐amino‐4‐cyano‐1H‐imidazol‐2‐yl)valinate. The crude residue was purified by silica gel chromatography eluting with Dietil ether/Hexane (6 : 1). ^1^H‐NMR (400 MHz, DMSO‐*d*6, ppm): δ 7.24 (s, 1H, NH), 6.40 (d, J=2.8 Hz, NH), 4.08‐4.04 (m, 1H, CH), 3.63 (S, 3H, O−CH_3_), 1.95–1.98 (m, 1H, CH); 0.87‐0.84 (m, 6H, CH_3_). ^13^C‐NMR (100 MHz, CDCl_3_, ppm): δ 174.31 (C=O), 158.84 (C), 140.08 (C), 120.89 (CN), 89.16(C), 58.04 (CH), 52.16 (O−CH_3_), 31.31 (CH), 19.03 (CH_3_), 17.71 (CH_3_). MS (ESI): m/z (M+H) +238.12. Elemental analysis for C_10_H_15_N_5_O_2_ calcd C, 50.62; H, 6.37; N, 29.52; O, 13.49. Found: C, 50.58; H, 6.34; N, 29.51; O, 13.49.

*Compound 4c* Methyl (5‐amino‐4‐cyano‐1H‐imidazol‐2‐yl)phenylalaninate. The crude residue was purified by silica gel chromatography eluting with Dietil ether/Hexane (6 : 1). ^1^H‐NMR (400 MHz, DMSO‐*d*6, ppm): δ 7.31–7.15 (m, 5H, CH−Ar x5), 6.49 (d, J=4 Hz, NH), 4.40–4.36 (m, 1H, CH), 3.59 (s, 3H, O−CH_3_), 2.99‐2.84 (m, 2H, CH_2_). ^13^C‐NMR (100 MHz, DMSO‐d6, ppm): δ 175.68 (C=O), 158.44 (C), 138.29 (C), 137.59 (C), 129.61 (C−Ar x2), 128.66 (C−Ar x2), 126.71 (C−Ar) 120.04 (CN), 89.73 (C−Ar) 56.12 (CH), 51.74 (O−CH_3_), 30.79 (CH_2_).MS (ESI): m/z (M+H) +286.12. Elemental analysis for C_14_H_15_N_5_O_2_ calcd C, 58.94; H, 5.30; N, 24.55; O, 11.22. Found: C, 58.92; H, 5.28; N, 24.52; O, 11.20.

*Compound 4d* Methyl (5‐amino‐4‐cyano‐1H‐imidazol‐2‐yl)tyrosinate. The crude residue was purified by silica gel chromatography eluting with Dietil ether/Hexane (6 : 1). ^1^H‐NMR (400 MHz, DMSO‐*d*6, ppm): δ 9.18 (s, 1H, NH), 6.95 (d, J=4 Hz, CH−Ar x2), 6.66 (d, J=4 Hz, CH−Ar x2), 5.60 (s, 1H, OH), 4.06–3.58 overlapped (m, 1H, CH) 3.58 (s, 3H, O−CH_3_), 2.79–2.66 (m,2H, CH_2_). ^13^C‐NMR (100 MHz, CDCl_3_, ppm): δ 175.84 (C=O), 168.23 (C), 156.28 (C−OH), 139.28 (C), 130.54 (C−Ar x2), 128.21 (C−Ar), 120,52 (CN), 115.43 (C−Ar x2), 97.48 (C−Ar) 56.34 (CH), 51.74 (O−CH_3_), 36.50 (CH_2_). MS (ESI): m/z (M+H) +302.12. Elemental analysis for C_14_H_15_N_5_O_3_ calcd C, 55.81; H, 5.02; N, 23.24; O, 15.93. Found: C, 55.79; H, 4.98; N, 23.20; O, 15.90.

*Compound 7a* Methyl (2,6‐diamino‐9H‐purin‐8‐yl)glycinate. ^1^H‐NMR (400 MHz, CDCl_3_, ppm): δ 8.12 (s, 1H, NH), 6.80 (s, 2H, NH_2_ ) 5.61 (s, 1H, NH), 4.61 (s, 1H, NH_2_), 4.03 (d, J=2.7 Hz, CH_2_), 3.79 (s, 3H, O−CH_3_).^13^C‐NMR (100 MHz, CDCl_3_, ppm): δ 161.15 (C=O), 158.06 (C−Ar), 155.78 (C−Ar), 145.83 (C−Ar), 135.20 (C−Ar), 129.50 (C−Ar), 63.92 (CH_2_) 52.38 (O−CH_3_). MS (ESI): m/z (M+H) +338.10. Elemental analysis for C_8_H_11_N_7_O_2_ calcd C, 40.51; H, 4.67; N, 41.33; O, 13.49. Found: C, 40.49; H, 4.64; N, 41.30; O, 13.44.

*Compound 7b* Methyl (2,6‐diamino‐9H‐purin‐8‐yl)valinate. ^1^H‐NMR (400 MHz, CDCl_3_, ppm): δ 7.69 (s, 1H,NH), 5.93 (s, 2H, NH_2_ ), 4.84 (s, 2H, NH_2_ ), 4.02 (d, J=1.8 Hz, CH), 3.84 (s, 3H, O−CH_3_ ), 3.75 (t, J=8.8 Hz, NH), 2.27–2.23 (m, 1H, CH), 1.07 (d, J=3.6 Hz, CH_3_ ), 0.97 (d, J=3.4 Hz, CH_3_ ). ^13^C‐NMR (100 MHz, CDCl_3_, ppm): δ 174.13 (C=O), 157.87 (C), 153.80 (C), 135.74 (C), 125.53 (C), 116.37 (C), 63.99 (CH), 51.90 (O−CH_3_), (C), 30.32 (CH), 18.75 (CH_3_), 16.09 (CH_3_). MS (ESI): *m*/*z* (M+H) +280.30. Elemental Analysis for C_11_H_17_N_7_O_2_ calcd C, 47.30; H, 6.67; N, 35.10; O, 11.46. Found: C, 40.45; H, 4.60; N, 41.28; O, 13.45.

*Compound 7c* Methyl (2,6‐diamino‐9H‐purin‐8‐yl)phenylalaninate. ^1^H‐NMR (400 MHz, CDCl_3_, ppm): δ 7.23–7.15 (m, 4 Hz, CH−Ar x3), 7.12 (d, J=3.4 Hz, CH−A x2r), 5,80 (s, 2H, NH_2_) 5.37 (d, J=3.7 Hz, NH_2_), 4.86–4.76 (m, 1H, CH), 3.74 (s, 3H, O−CH_3_), 3.12–3.09 (m, 2H, CH_2_). ^13^C‐NMR (100 MHz, DMSO‐*d*6, ppm): δ 175.65 (C=O), 173.53 (C), 158.42 (C), 157.58 (C), 137.62 (C), 136.09 (C), 130.19 (C), 129.59 (C−Ar x2), 128.73 (C−Ar), 128.55 (C−Ar), 127.12 (C−Ar), 126.99 (C), 58.84 (CH), 52.12 (O−CH_3_), 38.06 (CH_2_). MS (ESI): m/z (M+H) +328.14. Elemental analysis for C_15_H_17_N_7_O_2_ calcd C, 55.04; H, 5.23; N, 29.95; O, 9.77. Found: C, 54.50; H, 5.20; N, 29.90; O, 9.71.

*Compound 7d* Methyl (2,6‐diamino‐9H‐purin‐8‐yl)tyrosinate.^1^H‐NMR (400 MHz, CDCl_3_, ppm): 7.00 (d, J=4 Hz, CH−Ar x2), 6.77 (d, J=4 Hz, CH−Ar x2), 4.88 (d, J=4.4 Hz, NH), 4.76–3.55 (m, 1H, CH), 4.34–3.09 (m, 2H, CH_2_), 3.76 (s, 3H, O−CH_3_). ^13^C‐NMR (100 MHz, CDCl_3_, ppm): δ 177.91 (C=O), 162.57 (C),161,44 (C),155.11(C−Ar), 153.78 (C), 144.48 (C),143.80 (C−Ar), 135.44 (C−Ar x2), 130.92 (C−Ar), 119.39 (C−Ar x2), 67.61 (CH), 52.68 (O−CH_3_), 32.11 (CH_2_). MS (ESI): m/z (M+H) +344.14. Elemental analysis for C_15_H_17_N_7_O_3_ calcd C, 52.47; H, 4.99; N, 28.56; O, 13.98. Found: C, 52.42; H, 4.90; N, 28.51; O, 13.93.

*Compound 8a* (2,6‐diamino‐9H‐purin‐8‐yl)glycine ^1^H‐NMR (400 MHz, MeOD‐*d*4, ppm): δ 8.56 (s, 1H, NH), 8.13 (s, 1H, NH), 3.37 (s, 2H, CH_2_).MS (ESI): *m*/*z* (M+H) +224.08. Elemental analysis for C_7_H_9_N_7_O_2_ C, 37.67; H, 4.06; N, 43.93; O, 14.34. Found: C, 37.67; H, 4.06; N, 43.93; O, 14.34.

*Compound 8b (*2,6‐Diamino‐9H‐purin‐8‐yl)valine. ^1^H‐NMR (400 MHz, CDCl_3_, ppm): δ 6.99 (s, 1H, NH), 6.46 (S, 1H NH), 4.02 (s, 1H, OH), 4.78–3.73 (m, 1H, CH), 2.29–2.21 (m, 1H, CH), 1.08–0.96 (m, 6H, CH_3_). MS (ESI): m/z (M+H) +266.13. Elemental analysis for C_10_H_15_N_7_O_2_ C, 45.28; H, 5.70; N, 36.96; O, 12.06. Found: C, 44.90; H, 5.55; N, 35.91; O, 12.00.

*Compound 8c (*2,6‐Diamino‐9H‐purin‐8‐yl)phenylalanine. ^1^H‐NMR (400 MHz, MeOD‐*d*4, ppm): 7.29–7.23 (m, 5H, CH−Ar x5), 4.61 (s, 2H, OH), 4.39 (t, J=2.5 Hz, NH_2_), 3.37 (s, 1H, CH), 3.14–3.00 (m, 2H, CH_2_). MS (ESI): m/z (M+H) +314.13. Elemental analysis for C_14_H_15_N_7_O_2_ C, 53.67; H, 4.83; N, 31.29; O, 10.21. Found: C, 53.57; H, 4.43; N, 31.09; O, 10.01.

*Compound 8d* (2,6‐Diamino‐9H‐purin‐8‐yl)tyrosine. ^1^H‐NMR (400 MHz, MeOD‐*d*4, ppm): δ 8.56 (s, 1H, OH), 7.05 (d, J=4.2 Hz, CH−Ar x2), 6.71 (d, J=4.2 Hz, CH.Ar x2), 4.59 (s, OH), 4.34–4.31 (m, CH), 3.01–2.92 (m, 2H, CH_2_). MS (ESI): *m*/*z* (M+H) +330.12. Elemental analysis for C_14_H_15_N_7_O_3_ C, 51.06; H, 4.59; N, 29.77; O, 14.57. Found: C, 51.00; H, 4.51; N, 29.71; O, 14.51.

*Compound 9a* Methyl (6‐amino‐2‐oxo‐3,9‐dihydro‐2H‐purin‐8‐yl)glycinate. ^1^H‐NMR (400 MHz, CDCl_3_, ppm): δ 8.12 (s, 1H, NH), 7.00 (s, 1H, NH), 5.29 (d, J=0.8 Hz, NH_2_), 4.03 (d, J=2.6 Hz, CH_2_), 3.79 (s, 3H, O−CH_3_). ^13^C‐NMR (100 MHz, CDCl_3_, ppm): δ 165.06 (C=O), 160.75 (C=O), 153.98 (C), 148,7 (C), 141,98 (C), 129,56 (C), 67.83 (CH_2_), 52.23 (O−CH_3_). MS (ESI): *m*/*z* (M+H) +239.08. Elemental analysis for C_8_H_10_N_6_O_3_ C, 40.34; H, 4.23; N, 35.28; O, 20.15. Found: C, 40.31; H, 4.20; N, 35.22; O, 20.12.

*Compound 9b* Methyl (6‐amino‐2‐oxo‐3,9‐dihydro‐2H‐purin‐8‐yl)valinate. ^1^H‐NMR (400 MHz, CDCl_3_, ppm): δ 7.19 (s, 1H, NH), 6.24 (d, J=4.5 Hz, NH), 5.25 (s, 1H, NH_2_), 4.38–4.35 (m, 1H, CH), 3.73 (s, 3H, O−CH_3_), 2.14‐2.09 (m, 1H, CH), 0,95 (d, J=3.4 Hz, 3H, CH_3_), 0.88 (d, J=3.4 Hz, 3H, CH_3_). ^13^C‐NMR (100 MHz, CDCl_3_, ppm): δ 174.47 (C=O), 161.26 (C), 159.48 (C=O), 156.66 (C), 155.18 (C), 128.94 (C−Ar), 125.81 (C), 58.09 (CH), 52.14 (O−CH_3_), 19.02 (CH_3_), 17.69 (CH_3_). MS (ESI): *m*/*z* (M+H) +281.32. Elemental analysis for C_11_H_16_N_6_O_3_ C, 47.14; H, 5.75; N, 29.98; O, 17.12. Found: C, 47.11; H, 5.72; N, 29.94; O, 17.10.

*Compound 9c* Methyl (6‐amino‐2‐oxo‐3,9‐dihydro‐2H‐purin‐8‐yl)phenylalaninate. ^1^H‐NMR (400 MHz, CDCl_3_, ppm): δ 7.21 overlapped (*t*, CH−Ar), 7.20 (d, J=3.6 Hz, CH−Ar x2), 7.12 (d, J=3.6 Hz, CH−Ar x2), 5.79–4.74 (m, CH), 4.99 (s, 2H, NH_2_), 3.71 (s, 3H, O−CH_3_), 3.12–3.09 (m, 2H, CH_2_). ^13^C‐NMR (100 MHz, CDCl_3_, ppm): δ 174.00 (C=O), 173.43 (C), 158.15 (C), 157.16 (C=O), 136.09 (C−Ar) 135.37 (C), 129.37 (C−Ar), 129.23 (C−Ar), 128.94 (C), 128.52 (C−Ar), 127.44 (C−Ar), 127.02 (C), 59.94 (O−CH_3_), 54.03 (CH), 38.41 (CH_2_). MS (ESI): *m*/*z* (M+H) +329.13. Elemental analysis for C_15_H_16_N_6_O_3_ C, 54.87; H, 4.91; N, 25.60; O, 14.62. Found: C, 54.87; H, 4.91; N, 25.60; O, 14.62.

*Compound 9d* Methyl (6‐amino‐2‐oxo‐3,9‐dihydro‐2H‐purin‐8‐yl)tyrosinate. ^1^H‐NMR (400 MHz, DMSO‐*d*6, ppm): δ 8.10 (s, 2H, NH_2_), 7.05 (d, J=4.2 Hz, CH−Ar x2), 6.71 (d, J=4.2 Hz, CH.Ar x2), 5.60 (s, 1H, OH), 4.06–3.99 (m, 1H, CH) 3.58 (s, 3H, O−CH_3_), 2.96–2.80 (m,2H, CH_2_). ^13^C‐NMR (100 MHz, CDCl_3_, ppm): δ 171.75 (C=O), 166.89 (C), 164, 59 (C), 158.97 (C), 154.88 (C), 148.75 (C=O), 130.60 (C−Ar x2), 128.05 (C−Ar x2), 115.52 (C−Ar), 107.60 (C), 70.03 (CH), 51.33 (O−CH_3_), 29.57 (CH_2_). MS (ESI): *m*/*z* (M+H) +345.33. Elemental analysis for C_15_H_16_N_6_O_3_ C, 52.32; H, 4.68; N, 24.41; O, 18.59. Found: C, 54.814; H, 4.87; N, 25.40; O, 14.00.

*Compound 10a* (6‐Amino‐2‐oxo‐3,9‐dihydro‐2H‐purin‐8‐yl)glycine. ^1^H‐NMR (400 MHz, MeOD‐*d*4, ppm): δ 8.56 (s, 1H, NH), 8.13 (s, 1H, OH), 3.69 (S, 1H, NH), 3.37 (s, 1H, CH_2_). MS (ESI): *m*/*z* (M+H) +225.07. Elemental analysis for C_7_H_8_N_6_O_3_ C, 37.50; H, 3.60; N, 37.49; O, 21.41. Found: C, 36.80; H, 3.40; N, 37.41; O, 21.38.

*Compound 10b* (6‐Amino‐2‐oxo‐3,9‐dihydro‐2H‐purin‐8‐yl)valine. ^1^H‐NMR (400 MHz, MeOD‐*d*4, ppm): δ 3.99 (s, 1H, CH), 3.39 (s, 1H, NH), 2.15 (d, J=1.5 Hz, CH), 1.05 (d, J=3.4 Hz, CH_3_), 0,92 (d, J=3.4 Hz, CH_3_). MS (ESI): *m*/*z* (M+H) +267.11. Elemental analysis for C_10_H_14_N_6_O_3_ C, 45.11; H, 5.30; N, 31.56; O, 18.03. Found: C, 45.01; H, 5.22; N, 31.50; O, 17.02.

*Compound 10c* (6‐Amino‐2‐oxo‐3,9‐dihydro‐2H‐purin‐8‐yl)phenylalanine. ^1^H‐NMR (400 MHz, MeOD‐*d*4, ppm): δ, 7.31–7.23 (m, 5H, CH−Ar x5), 4.39 (t, J=5 Hz, CH), 3.14–3.01 (m, 2H, CH_2_). MS (ESI): *m*/*z* (M+H) +315.11. Elemental analysis for C_14_H_14_N_6_O_3_ C, 53.50; H, 4.49; N, 26.74; O, 15.25. Found: C, 53.40; H, 4.49; N, 26.72; O, 15.21.

*Compound 10d* (6‐Amino‐2‐oxo‐3,9‐dihydro‐2H‐purin‐8‐yl)tyrosine. ^1^H‐NMR (400 MHz, MeOD‐*d*4, ppm): δ, 7.05 (d, J=4.2 Hz, CH−Ar x2), 6.71 (d, J=4.2 Hz, CH−Ar x2), 4.32 (t, J=5 Hz, CH), 3.00–2.92 (m, 2H, CH_2_). MS (ESI): *m*/*z* (M+H) +331.11. Elemental analysis for C_14_H_14_N_6_O_4_ C, 50.91; H, 4.27; N, 25.44; O, 19.37. Found: C, 50.89; H, 4.22; N, 25.41; O, 19.32.

*Compound 11a* Methyl (6‐oxo‐6,9‐dihydro‐3H‐purin‐8‐yl)glycinate. ^1^H‐NMR (400 MHz, CDCl_3_, ppm): δ 8.57 (s, 1H, NH), 8.36 (s, 1H, CH), 4.11 (d, J=2.8 Hz, CH_2_), 3.80 (s, 3H, O−CH_3_). ^13^C‐NMR (100 MHz, CDCl_3_, ppm): δ 169.55 (C=O), 161.55 (C), 161.06 (C=O), 153.31 (C), 147.87 (C), 124.96 (C), 52.48 (O−CH_3_), 31.92 (CH_2_). MS (ESI): *m*/*z* (M+H) +224.07. Elemental analysis for C_8_H_9_N_5_O_3_ C, 43.05; H, 4.06; N, 31.38; O, 21.50. Found: C, 43.00; H, 4.01; N, 31.35; O, 21.54.

*Compound 11b* Methyl (6‐oxo‐6,9‐dihydro‐3H‐purin‐8‐yl)valinate^. 1^H‐NMR (400 MHz, CDCl_3_, ppm): δ 8.60 (s, 1H, NH), 8.38 (s, 1H, CH−Ar), 5.93 (s, 1H, NH), 4.43 (s, 1H, CH), 3.76 (s, 3H, O−CH_3_), 2.16–2.11 (m, 1H, CH), 0.97‐0.90 (m, 6H, CH_3_). ^13^C‐NMR (100 MHz, CDCl_3_, ppm): δ 173.85 (C=O), 164.61 (C), 161.67 (C), 157.35 (C=O), 146.97 (C), 131.37 (C), 58.11 (C), 52.11 (O−CH_3_), 31.40 (C), 18.98 (CH_3_), 17.74 (CH_3_). MS (ESI): *m*/*z* (M+H) +266.12. Elemental analysis for C_11_H_15_N_5_O_3_ C, 49.81; H, 5.70; N, 26.40; O, 18.09. Found: C, 49.80; H, 5.65; N, 26.42; O, 18.00.

*Compound 11c* Methyl (6‐oxo‐6,9‐dihydro‐3H‐purin‐8‐yl)phenylalaninate ^1^H‐NMR (400 MHz, MeOD‐*d*4, ppm): δ 8.04 (s, 1H, CH−Ar), 7.31–7.25 (m, CH−Ar x5), 4.39 (T, J=5.2 Hz, CH), 3.36 (s, 1H, O−CH_3_), 3.14–3.01 (m, 2H, CH_2_). ^13^C‐NMR (100 MHz, CDCl_3_, ppm): δ 180.66 (C=O), 173.88 (C), 163.70 (C), 158.30 (C=O), 153.53 (C) 129.43 (C−Ar x2), 128.78 (C−Ar x2), 127.92 (C−Ar), 112.17 (C−Ar), 100.62 (C), 53.46 (O−CH_3_), 33.77 (CH_2_). MS (ESI): m/z (M+H) +314.12. Elemental analysis for C_15_H_15_N_5_O_3_ C, 57.50; H, 4.83; N, 22.35; O, 15.32 Found: C, 49.80 H, 5.66; N, 26.44; O, 18.02.

*Compound 11d* Methyl (6‐oxo‐6,9‐dihydro‐3H‐purin‐8‐yl)tyrosinate. ^1^H‐NMR (400 MHz, MeOD‐*d*4, ppm): δ 8.37 (s, 1H, CH−Ar), 7.20 (d, J=4 Hz, CH−Ar x2), 6.85 (d, J=4 Hz, CH−Ar x2), 5.70 (s, 1H, OH), 4.36–4.30 (m,1H, CH) 3.36 (s, 1H, O−CH_3_), 3.06–2.90 (m, 2H, CH_2_). ^13^C‐NMR (100 MHz, CDCl_3_, ppm): δ 172.26 (C=O), 162.81 (C),161,27 (C),156.67 (C), 154.88 (C), 147.98 (C=O), 130.86 (C−Ar x2), 128.81 (C−Ar x2), 115.52 (C−Ar), 70.79 (CH), 57.91 (O−CH_3_), 37.11 (CH_2_). MS (ESI): m/z (M+H) +330.11. Elemental analysis for C_15_H_15_N_5_O_4_ C, 54.71; H, 4.59; N, 21.27; O, 19.43. Found: C, 49.80; H, 5.60; N, 26.32; O, 18.07.

*Compound 12a* (6‐oxo‐6,9‐dihydro‐3H‐purin‐8‐yl)glycine. ^1^H‐NMR (400 MHz, MeOD‐*d*4, ppm): δ 8.56 (s, 1H, CH), 3.69 (s, 2H, CH_2_). MS (ESI): *m*/*z* (M+H) +210.05. Elemental analysis for C_7_H_7_N_5_O_3_ C, 40.20; H, 3.37; N, 33.48; O, 22.95. Found: C, 40.15; H, 3.31; N, 33.42; O, 22.90.

*Compound 12b* (6‐oxo‐6,9‐dihydro‐3H‐purin‐8‐yl)valine. ^1^H‐NMR (400 MHz, MeOD‐*d*4, ppm): δ 8.56 (s, 1H, CH), 4.59 (s, 1H, NH), 4.02 (s, 1H, CH), 2.17–2.09 (m, 1H, CH),, 0.97–0.91 (m, 6H, 2×CH_3_).MS (ESI): *m*/*z* (M+H) +252.25. Elemental analysis for C_10_H_13_N_5_O_3_ C, 47.81; H, 5.22; N, 27.88; O, 19.10. Found: C, 47.77; H, 5.20; N, 27.81; O, 19.08.

*Compound 12c (*6‐oxo‐6,9‐dihydro‐3H‐purin‐8‐yl)phenylalanine. ^1^H‐NMR (400 MHz, CDCl_3_, ppm): δ 9.09 (s, 1H, CH−Ar), 7.34‐7.28 (m, CH−Ar x3), 7.09 (d, J=1.8 Hz., 2H, CH−Ar), 4.81 (d, J=1.3 Hz, CH), 3.64–3.34 (m, 2H, CH_2_). MS (ESI): *m*/*z* (M+H) +300.10. Elemental analysis for C_14_H_13_N_5_O_3_ C, 56.18; H, 4.38; N, 23.40; O, 16.04. Found: C, 56.12; H, 4.34; N, 23.35; O, 16.00.

*Compound 12d* (6‐oxo‐6,9‐dihydro‐3H‐purin‐8‐yl)tyrosine. ^1^H‐NMR (400 MHz, MeOD‐*d*4, ppm): δ 8.37 (s, 1H, CH−Ar), 7.95 (s, 1H, OH), 7.05 (d, J=4 Hz, CH−Ar x2), 6.77 (d, J=4 Hz, CH−Ar x2), 4.40–4.39 (m,1H, CH), 3.06–2.90 (m, 2H, CH_2_).

MS (ESI): *m*/*z* (M+H) +316.10. Elemental analysis for C_14_H_13_N_5_O_4_ C, 53.33; H, 4.16; N, 22.21; O, 20.30. Found: C, 53.31; H, 4.18; N, 22.20; O, 20.15.

## Supporting Information

Supporting information materials contains: SI#1: Spectroscopic data, ^1^H‐NMR and ^13^C‐NMR of **4 a**–**4 d**; **7 a**–**d**; **8 a**–**d**; **9 a**–**d**; **10 a**–**d**; **11 a**–**d**; **12 a**–**d** and SI#2: Experimental details of two component reactions SI#3: Chromatographic profile of reactions under complete prebiotic conditions.

## Conflict of Interests

The authors declare no conflict of interest.

1

## Supporting information

As a service to our authors and readers, this journal provides supporting information supplied by the authors. Such materials are peer reviewed and may be re‐organized for online delivery, but are not copy‐edited or typeset. Technical support issues arising from supporting information (other than missing files) should be addressed to the authors.

Supporting Information

## Data Availability

The data that support the findings of this study are available from the corresponding author upon reasonable request.
