# Supplementary material for: Multicomponent Synthesis of C(8)‐Substituted Purine Building Blocks of Peptide Nucleic Acids from Prebiotic Compounds
Source: ChemistryOpen. 2024 Oct 17;13(12):e202400265. doi: 10.1002/open.202400265 (PMC12056921; doi:10.1002/open.202400265)
Supplement: Supplementary file 1 — Supporting Information [file OPEN-13-e202400265-s001.pdf]

ChemistryOpen

Supporting Information

Multicomponent Synthesis of C(8)-Substituted Purine Building Blocks of Peptide Nucleic Acids from Prebiotic Compounds

Eleonora Mancin, Eliana Capecchi, Lorenzo Botta, and Bruno Mattia Bizzarri*

Supporting information

SI#1: 1H-NMR and 13C-NMR spectra of compounds **4a-d**, **7a-d**, **8a-d**, **9a-d**, **10a-d**, **11a-d** and **12a-d**.

SI#2: Experimental details of two component reactions

SI#3:. Chromatographic profile of reactions under complete prebiotic conditions.

SI#1: 1H-NMR and 13C-NMR spectra of compounds **4a-d**, **7a-d**, **8a-d**, **9a-d**, **10a-d**, **11a-d** and **12a-d**.

Compound **4a**

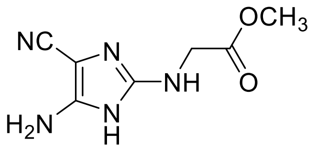

^{1}H-NMR

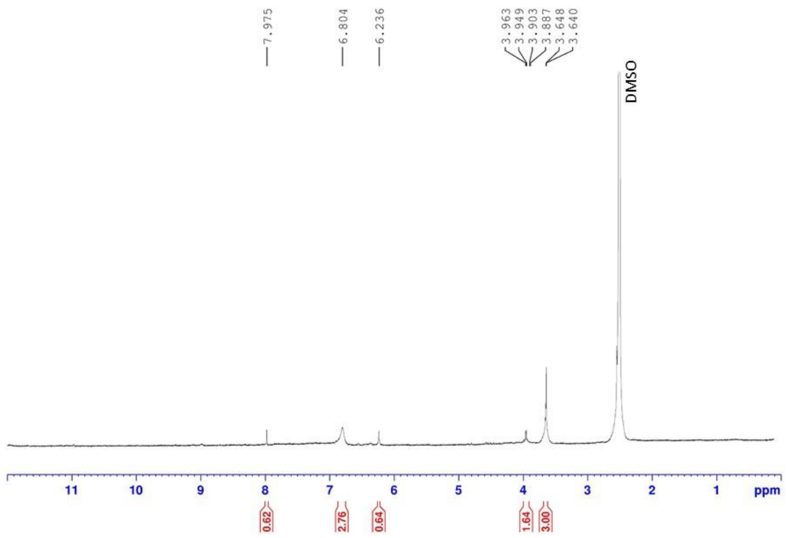

^{13}C-NMR

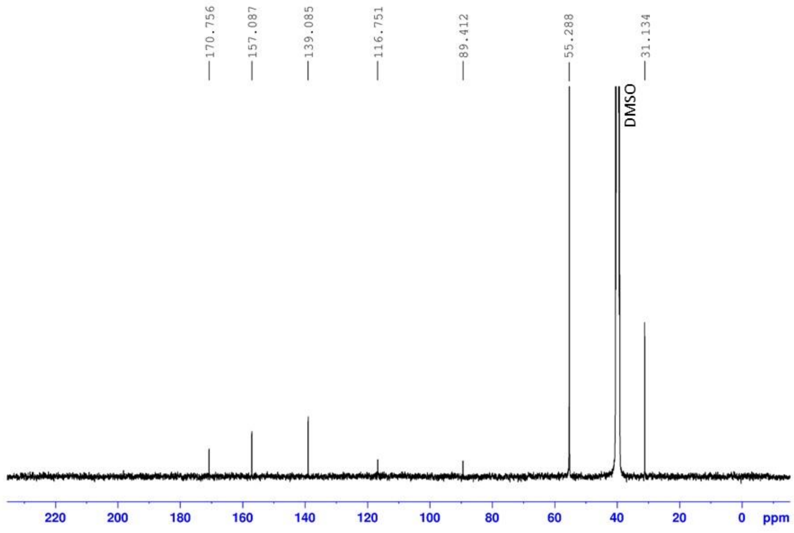

Supporting information

Compound **4b**

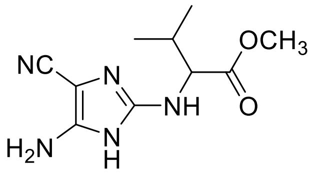

^{1}H-NMR

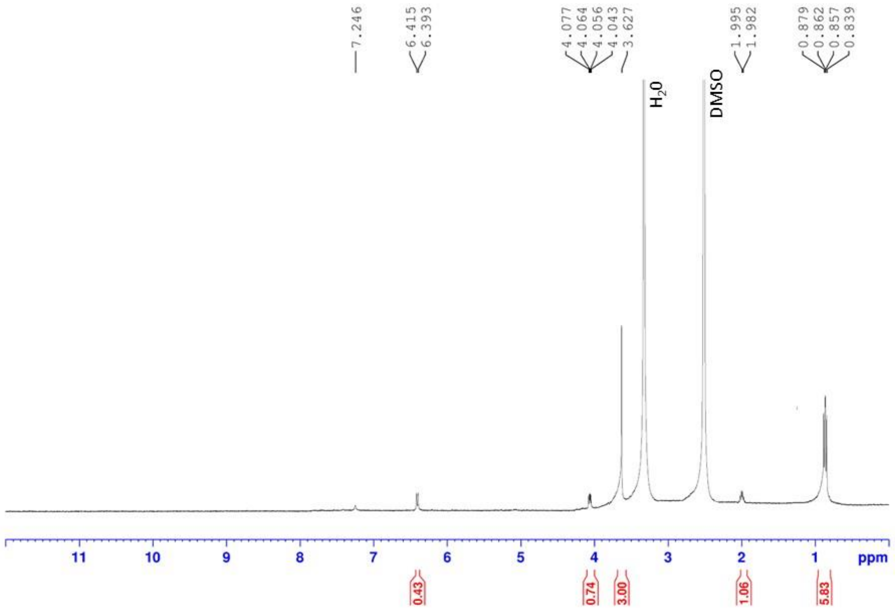

^{13}C-NMR

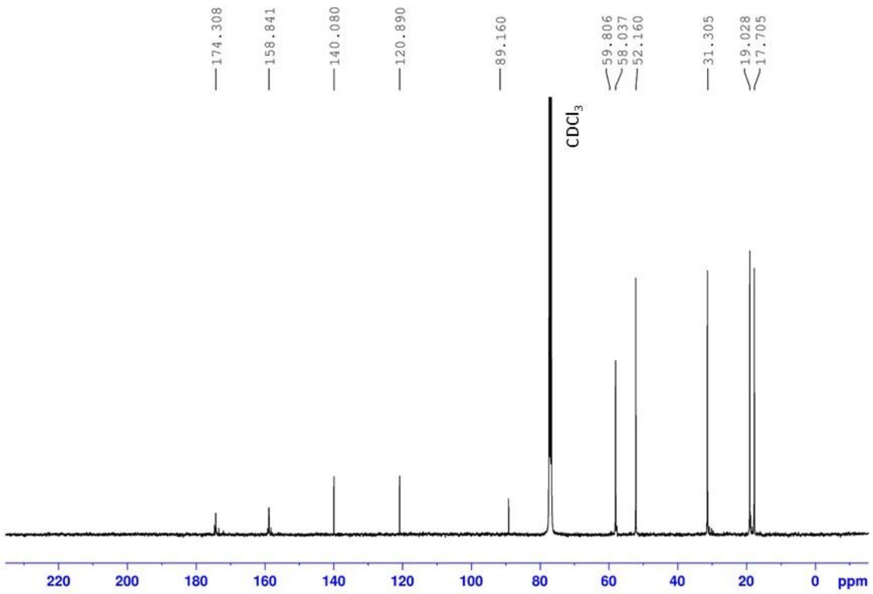

Supporting information

Compound **4c**

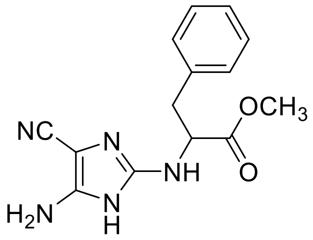

^{1}H-NMR

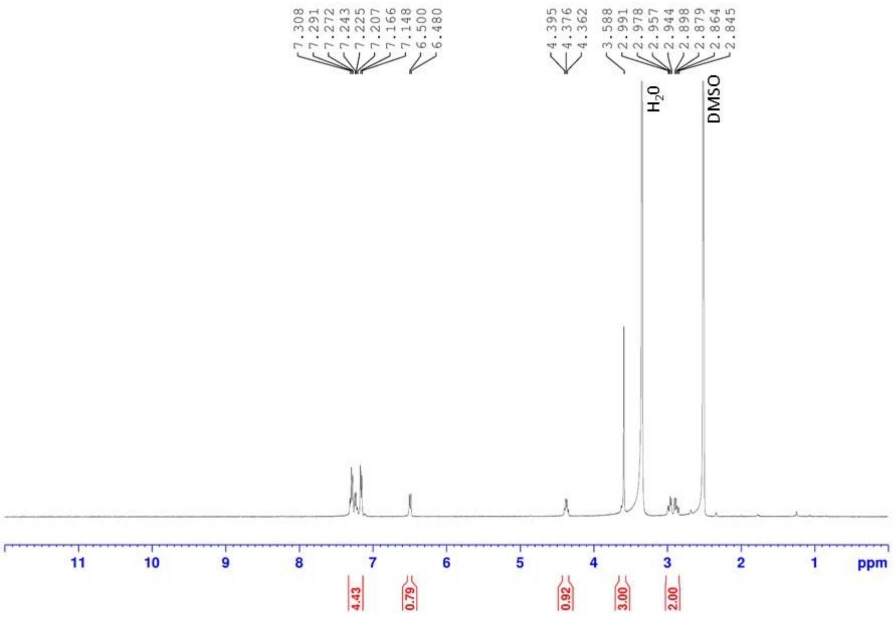

^{13}C-NMR

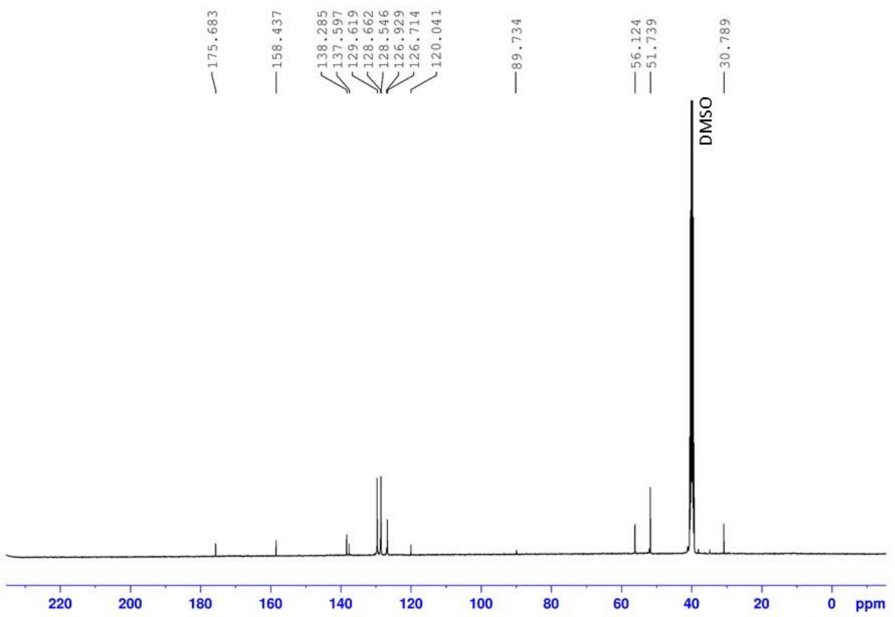

Supporting information

Compound **4d**

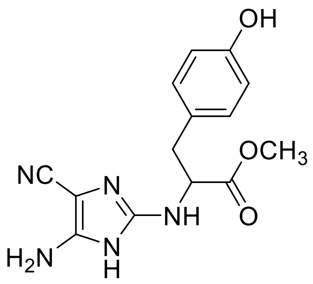

^{1}H-NMR

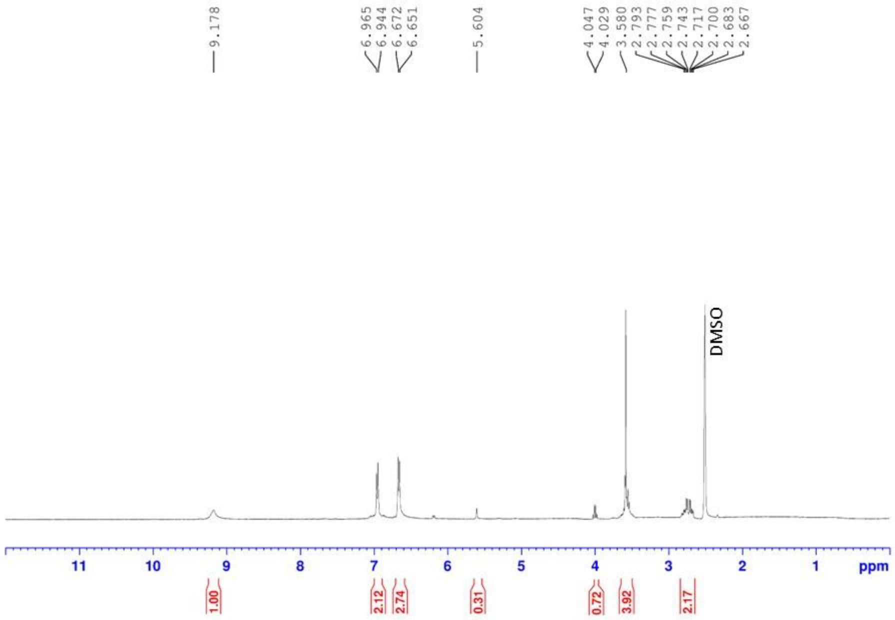

^{13}C-NMR

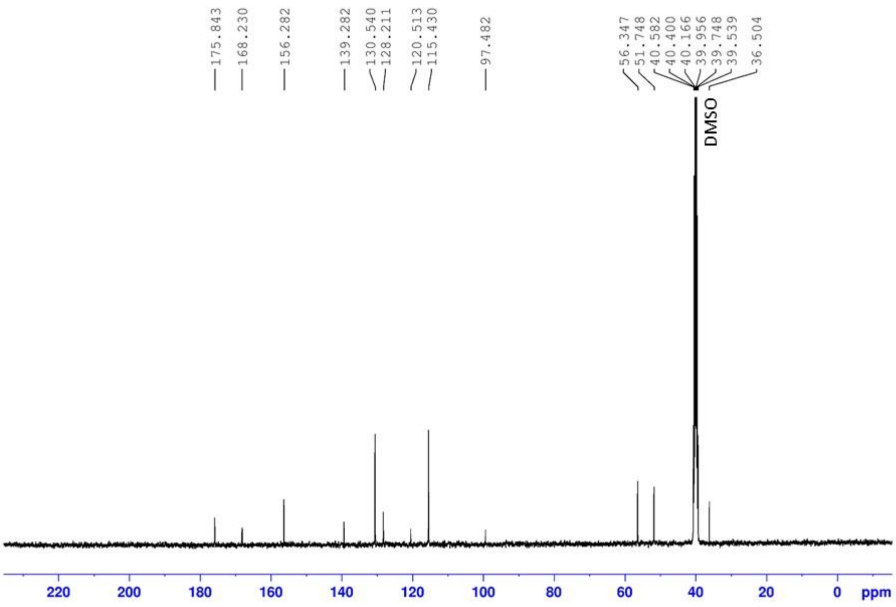

Supporting information

Compound **7a**

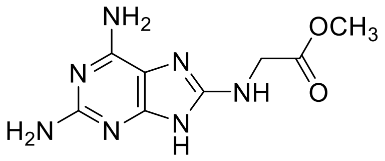

^{1}H-NMR

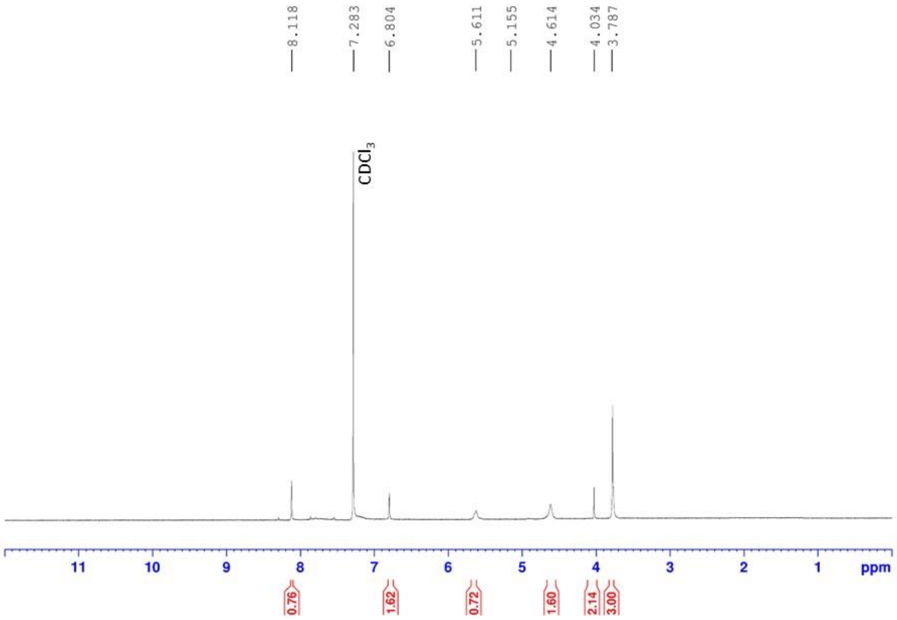

^{13}C-NMR

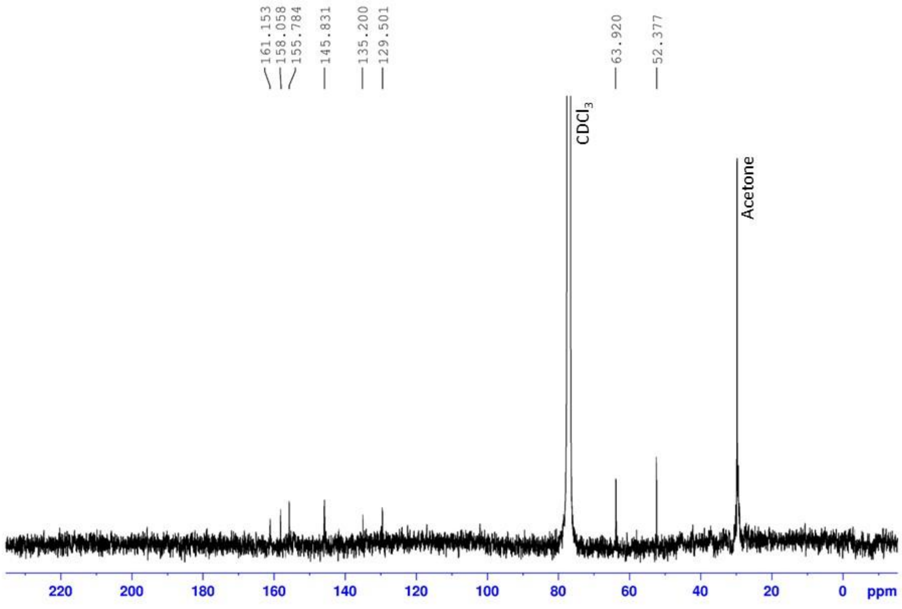

Supporting information

Compound **7b**

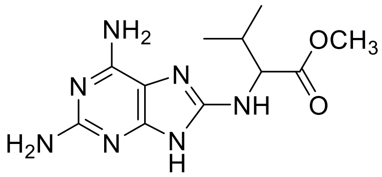

^{1}H-NMR

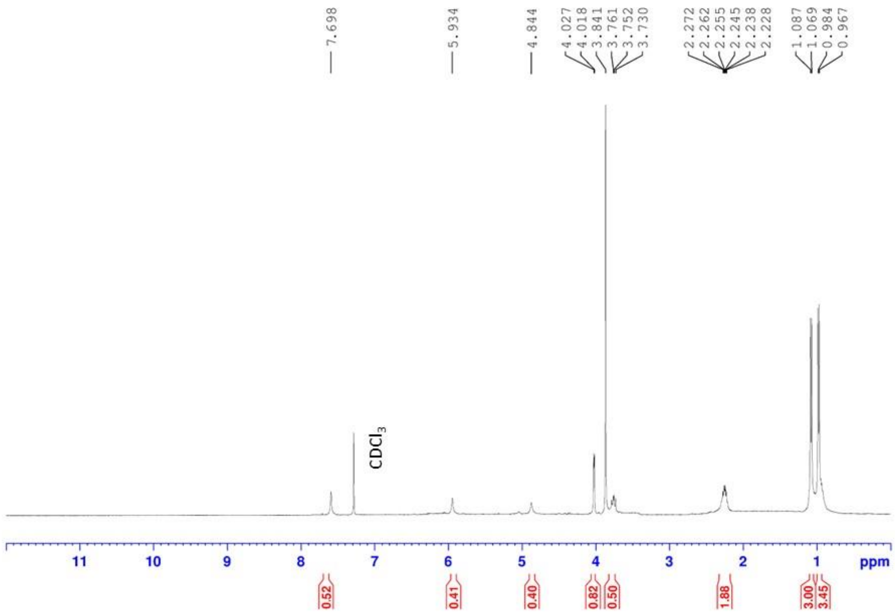

^{13}C-NMR

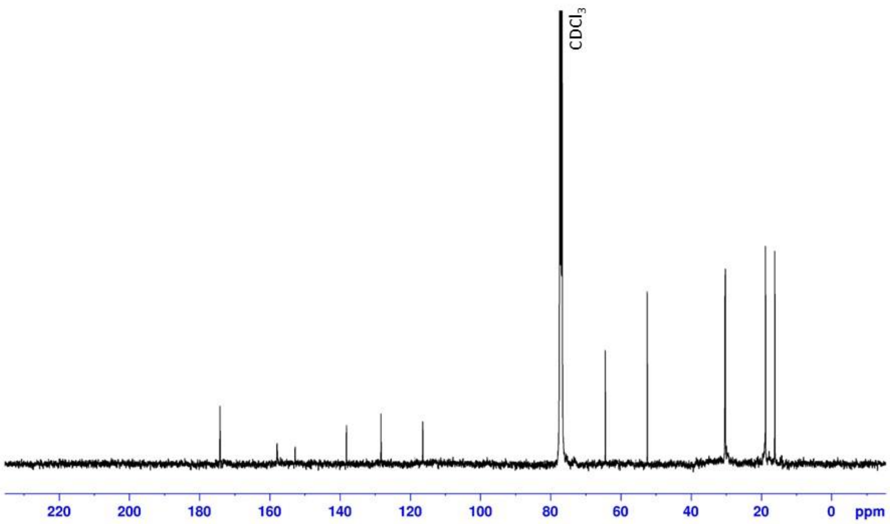

Supporting information

Compound **7c**

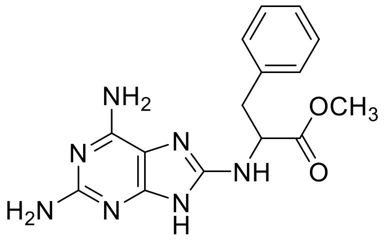

^{1}H-NMR

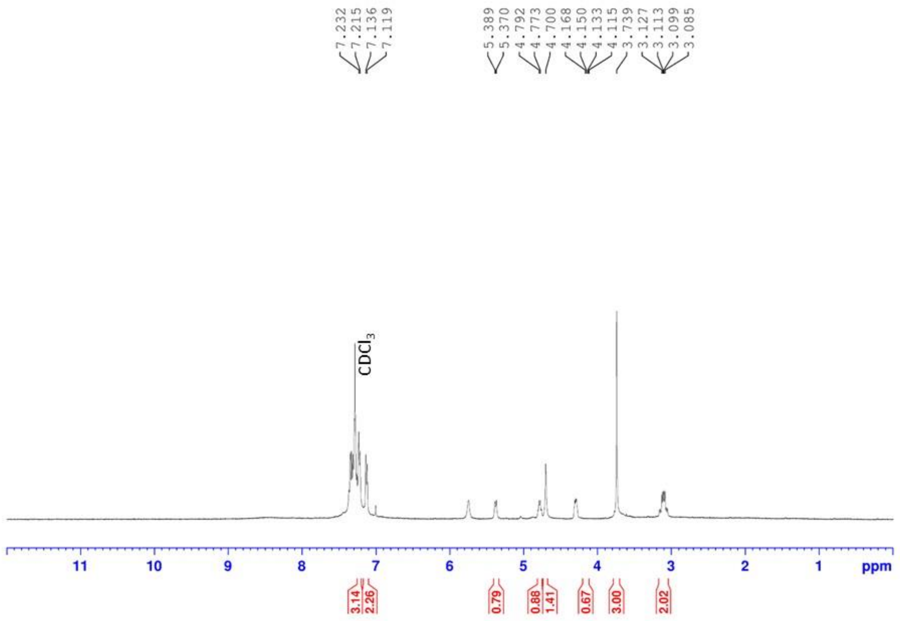

^{13}C-NMR

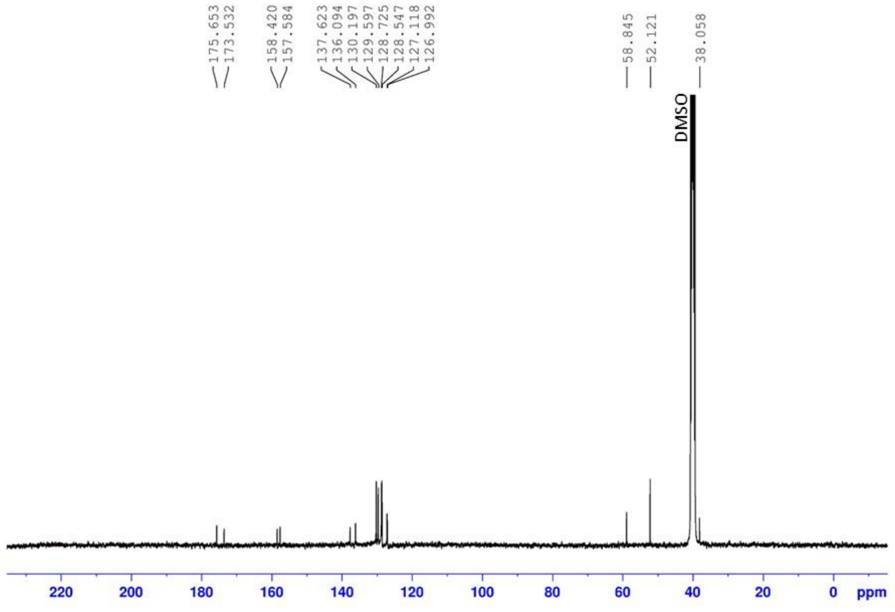

Supporting information

Compound **7d**

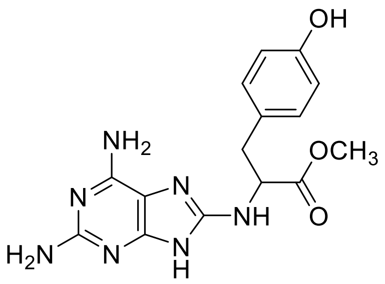

^{1}H-NMR

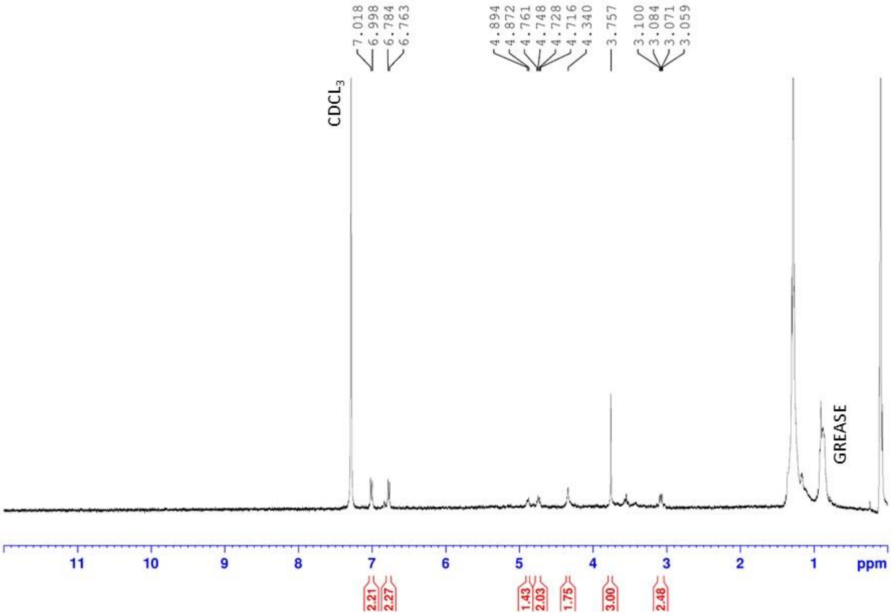

^{13}C-NMR

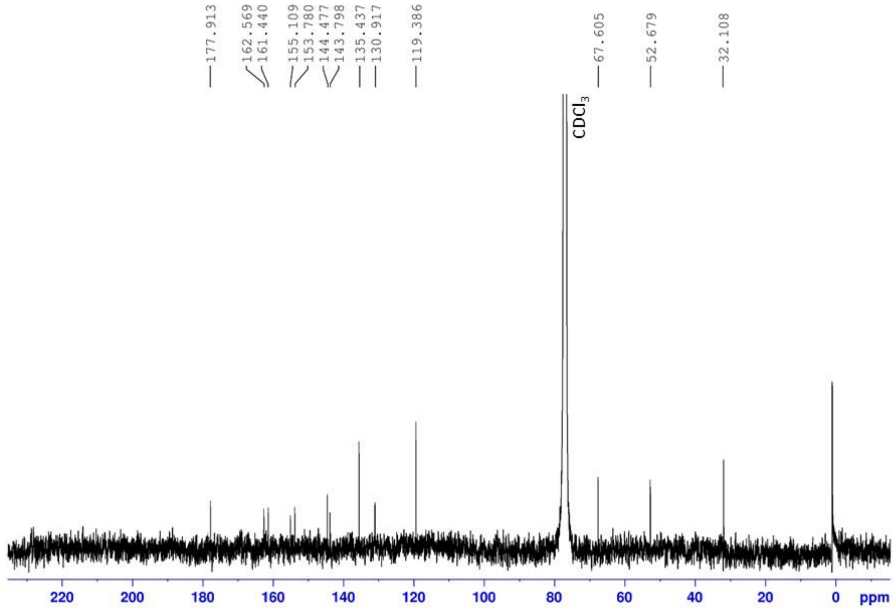

Supporting information

Compound **8a**

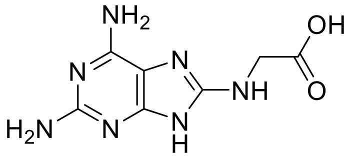

^{1}H-NMR

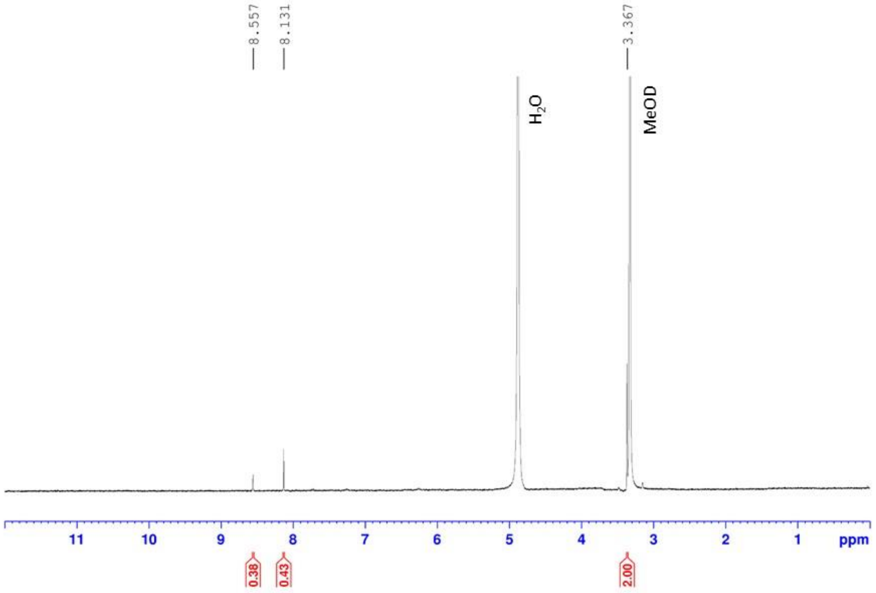

Compound **8b**

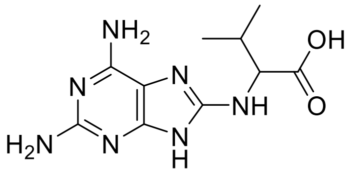

Supporting information

^{1}H-NMR

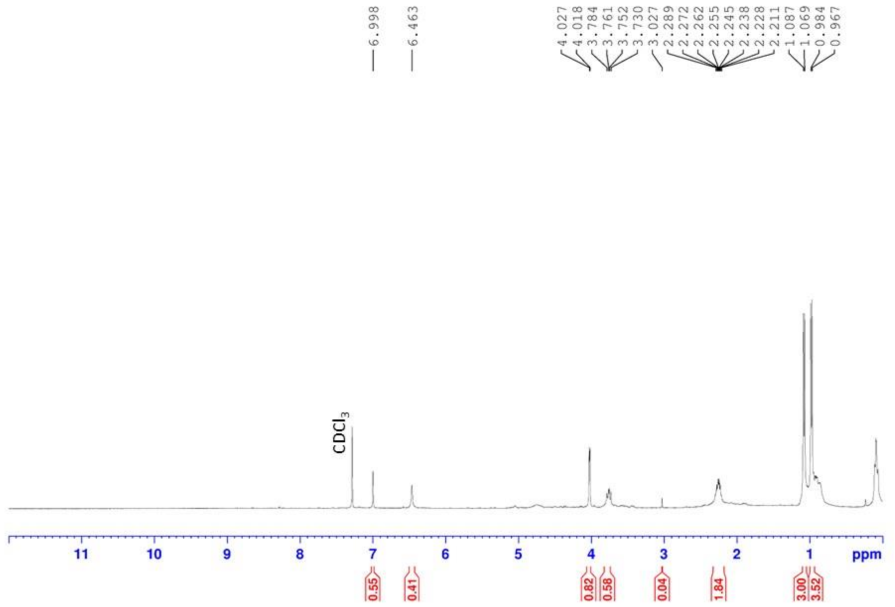

Compound **8c**

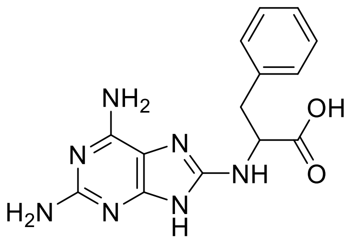

^{1}H-NMR

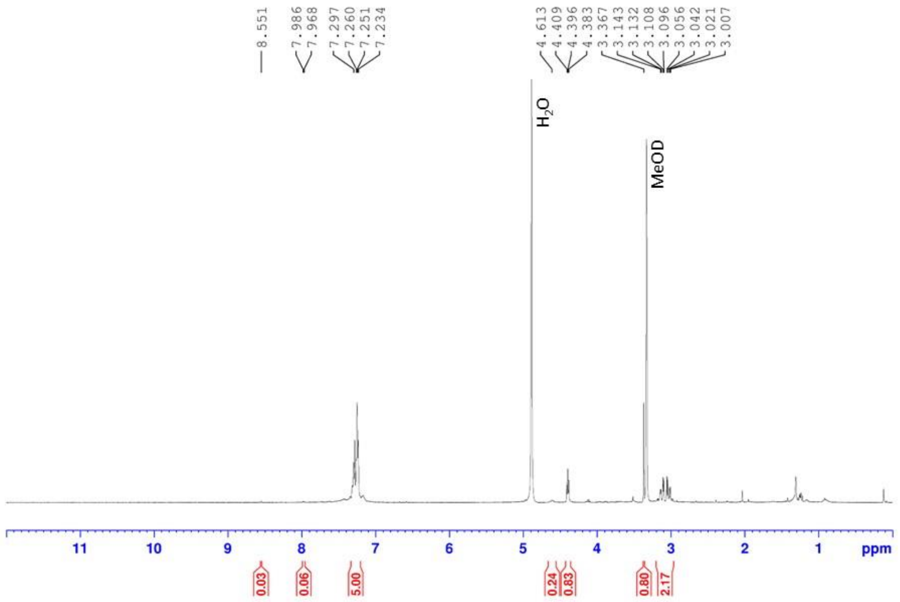

Supporting information

Compound **8d**

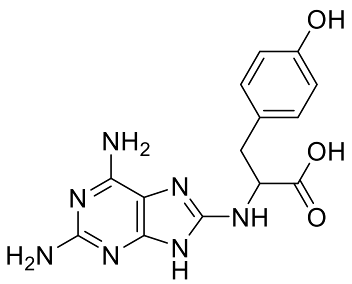

^{1}H-NMR

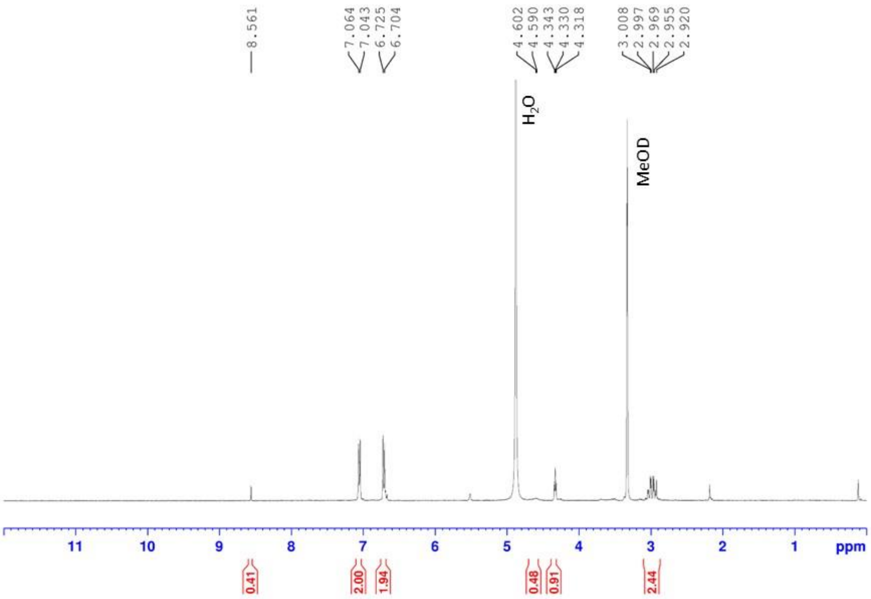

Compound **9a**

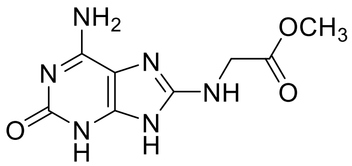

Supporting information

^{1}H-NMR

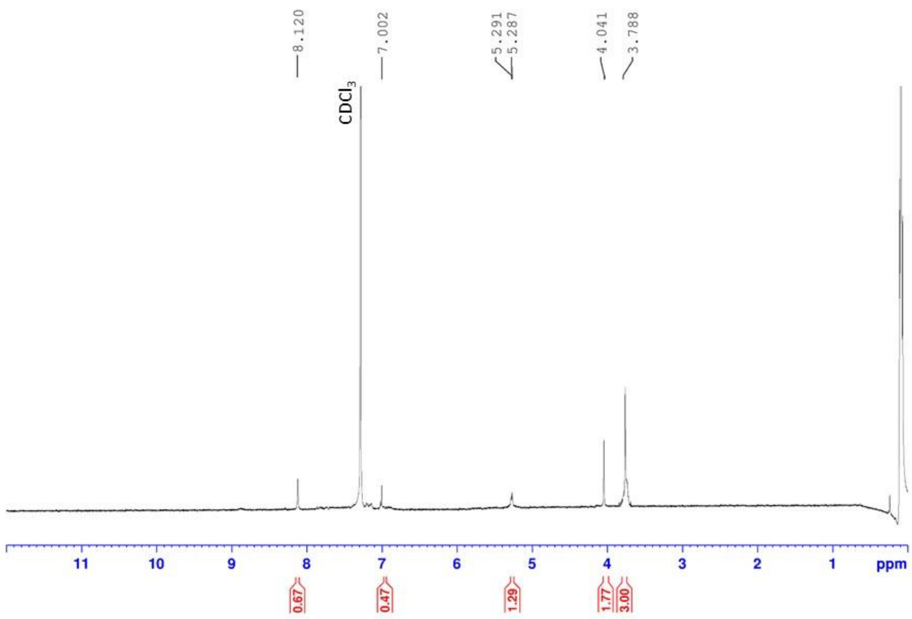

^{13}C-NMR

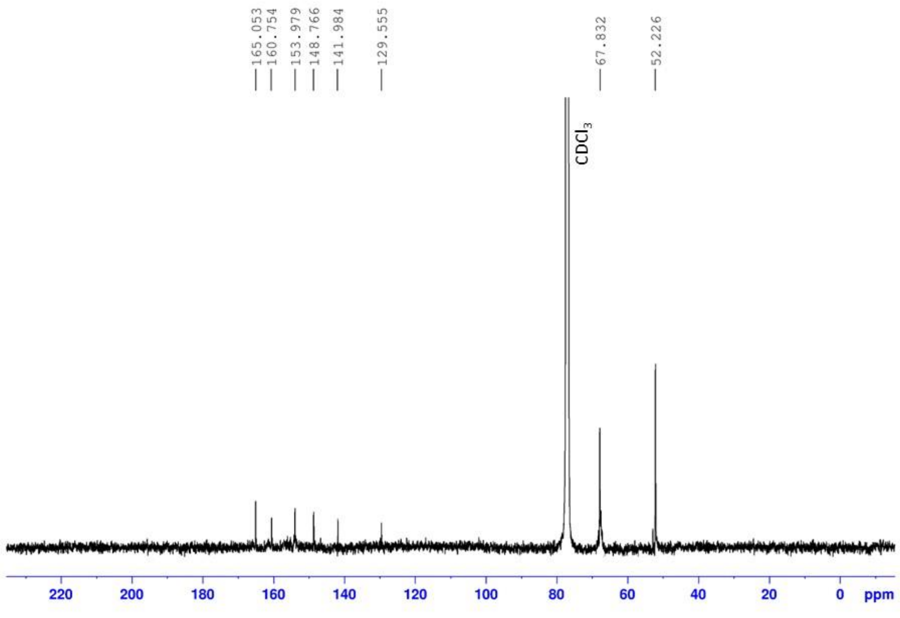

Supporting information

Compound **9b**

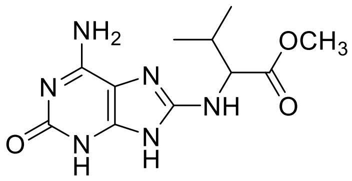

^{1}H-NMR

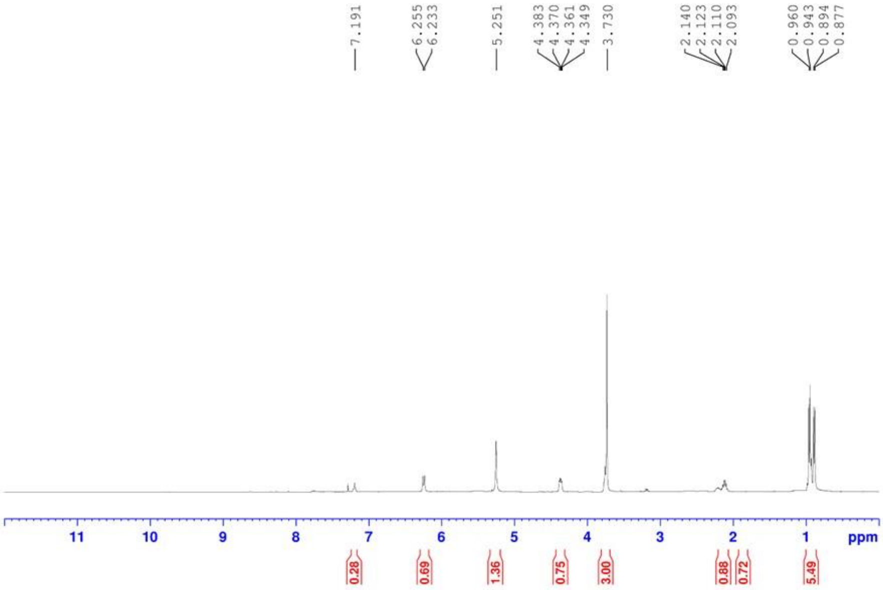

^{13}C-NMR

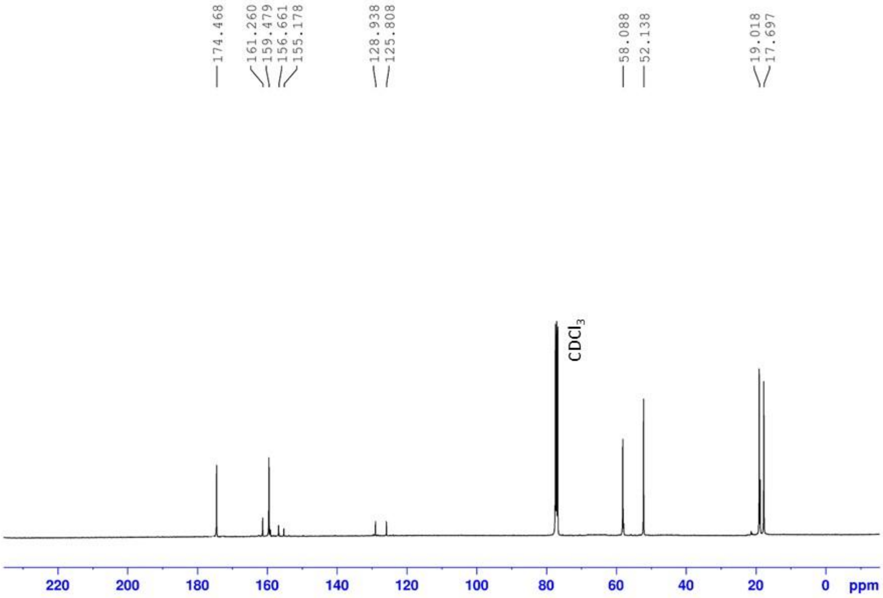

Supporting information

Compound **9c**

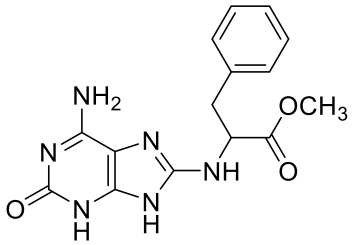

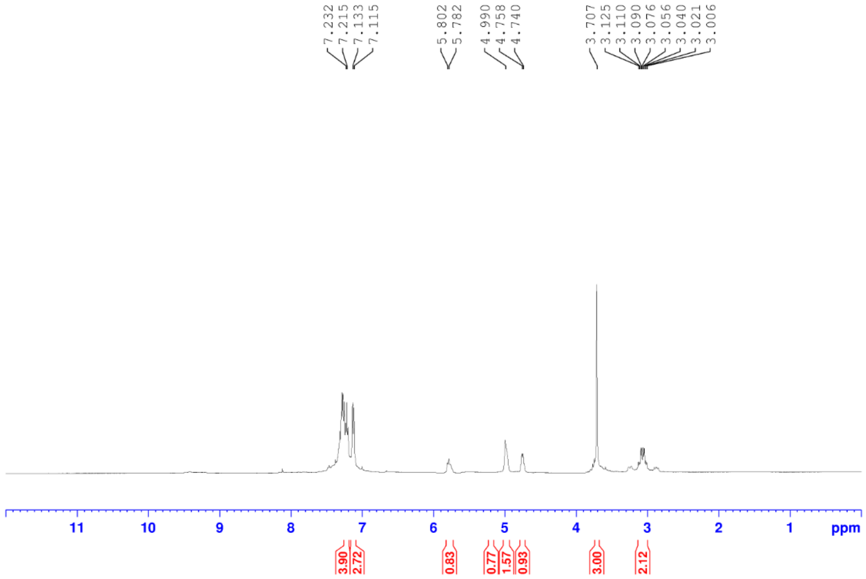

^{13}C-NMR

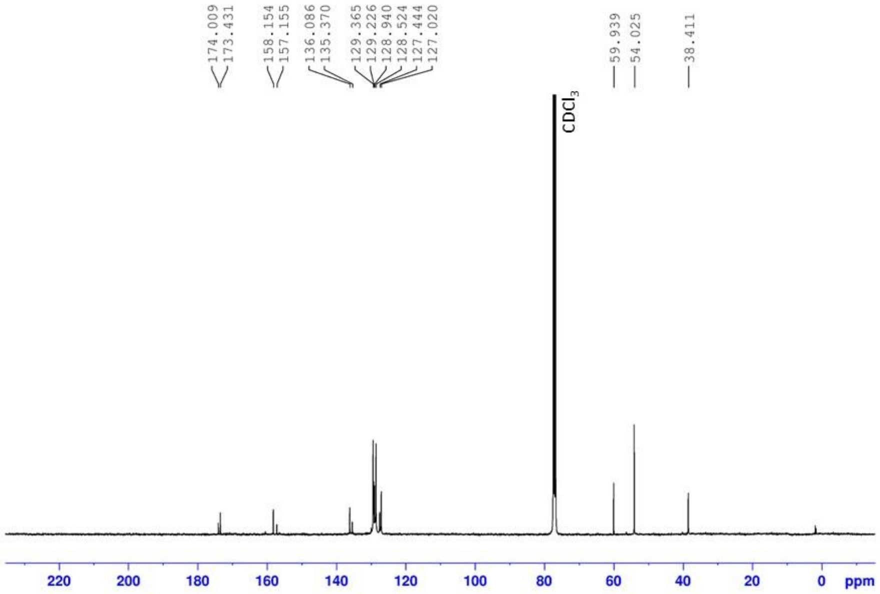

Supporting information

Compound **9d**

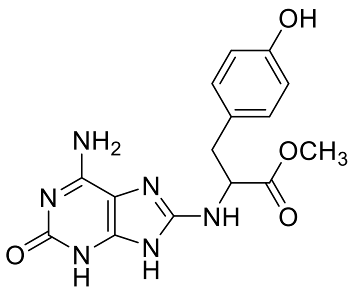

^{1}H-NMR

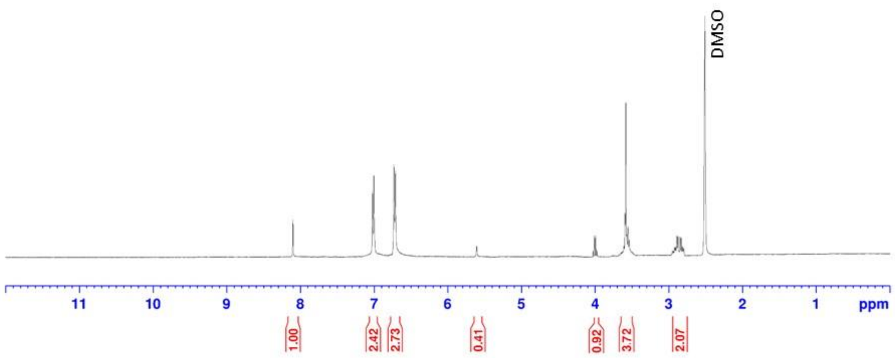

^{13}C-NMR

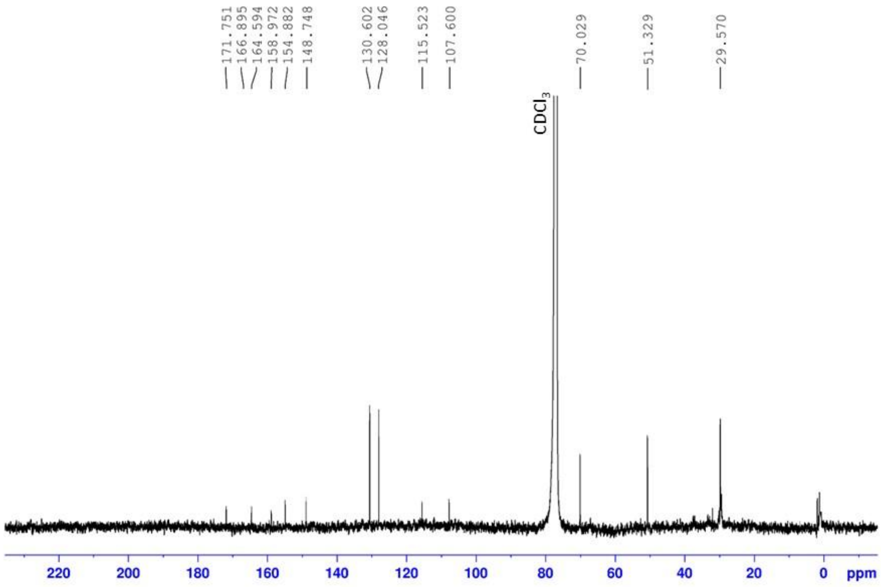

Supporting information

Compound **10a**

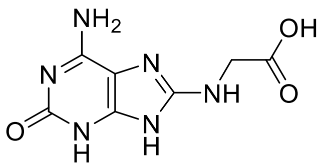

^{1}H-NMR

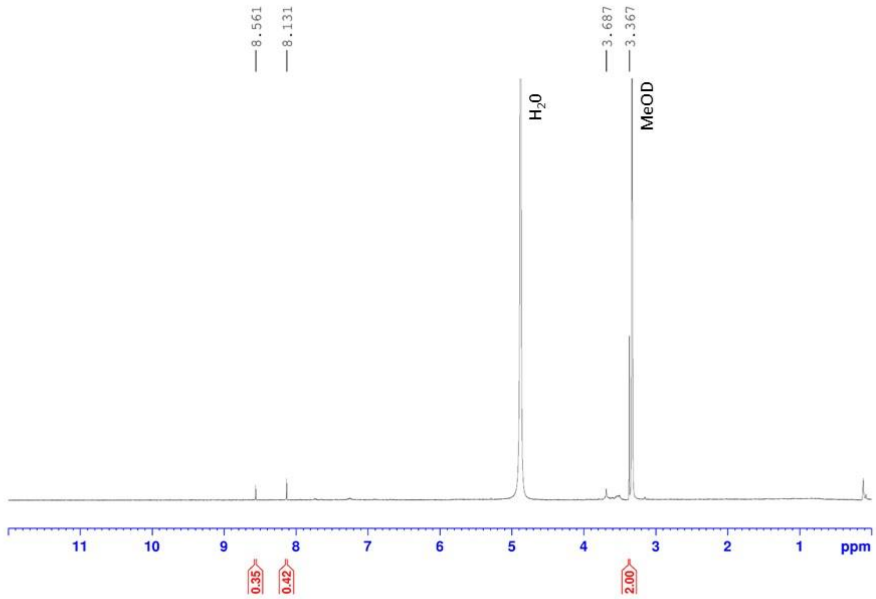

Compound **10b**

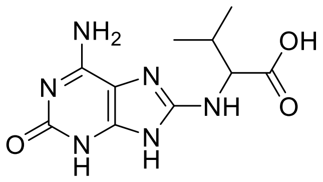

Supporting information

^{1}H-NMR

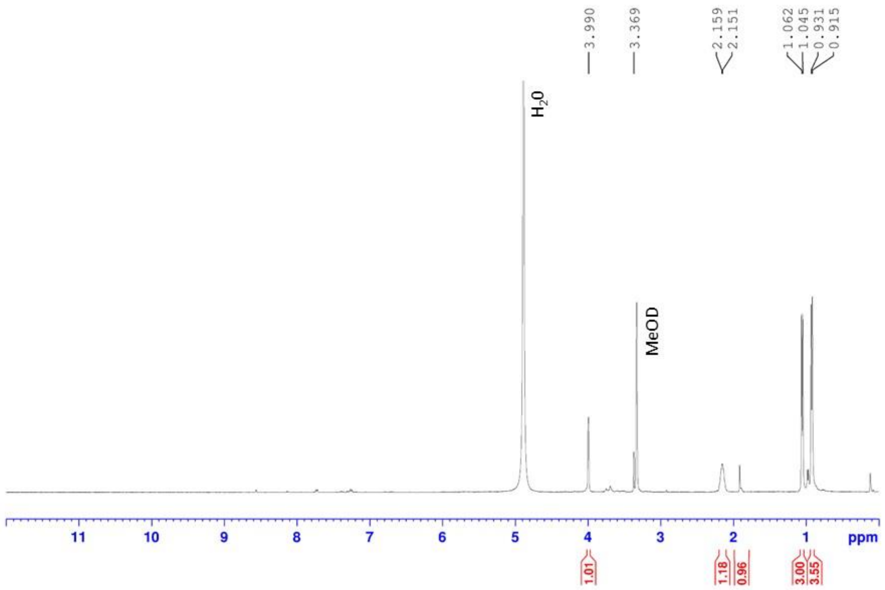

Compound **10c**

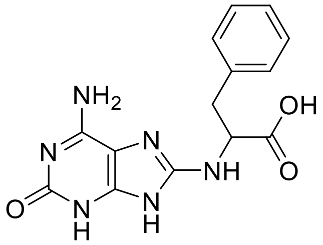

^{1}H-NMR

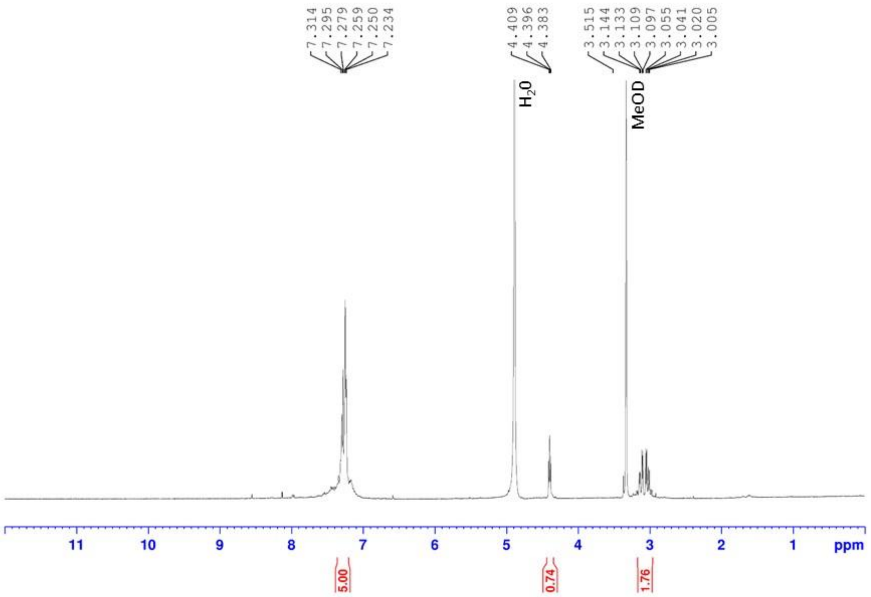

Supporting information

Compound **10d**

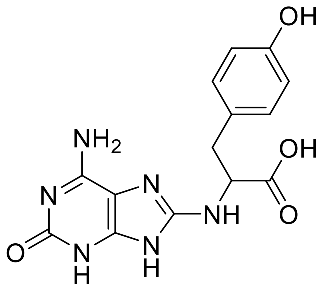

^{1}H-NMR

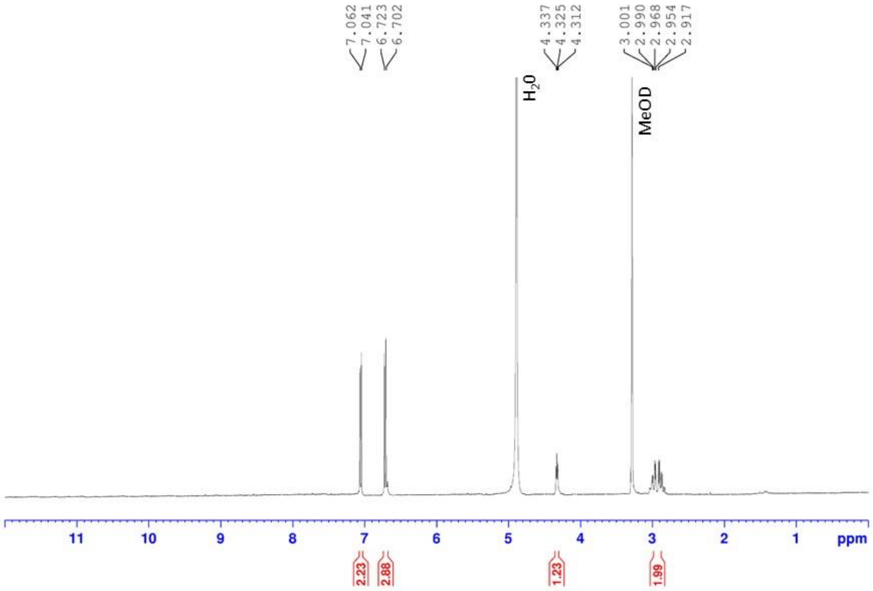

Compound **11a**

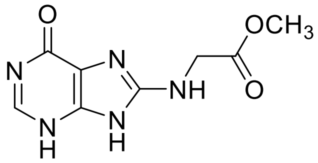

Supporting information

^{1}H-NMR

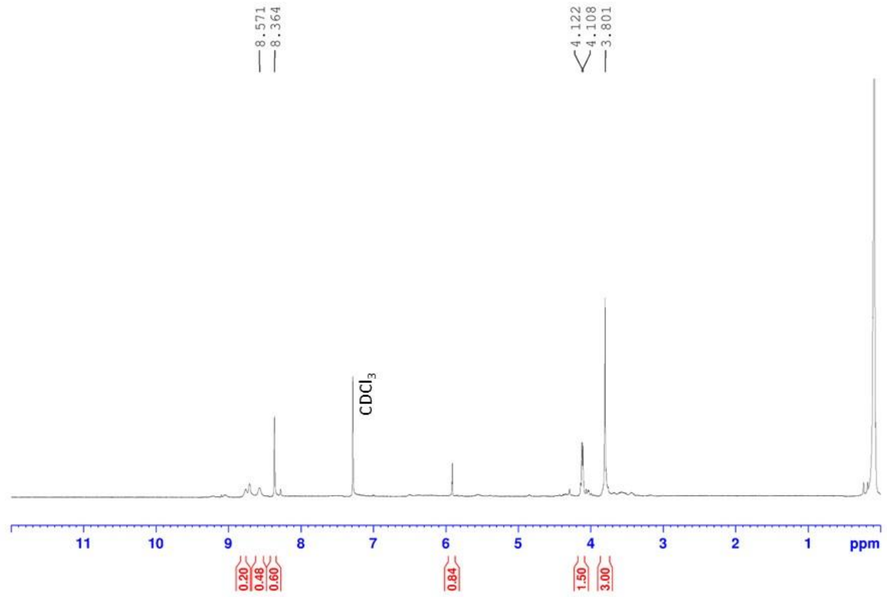

^{13}C-NMR

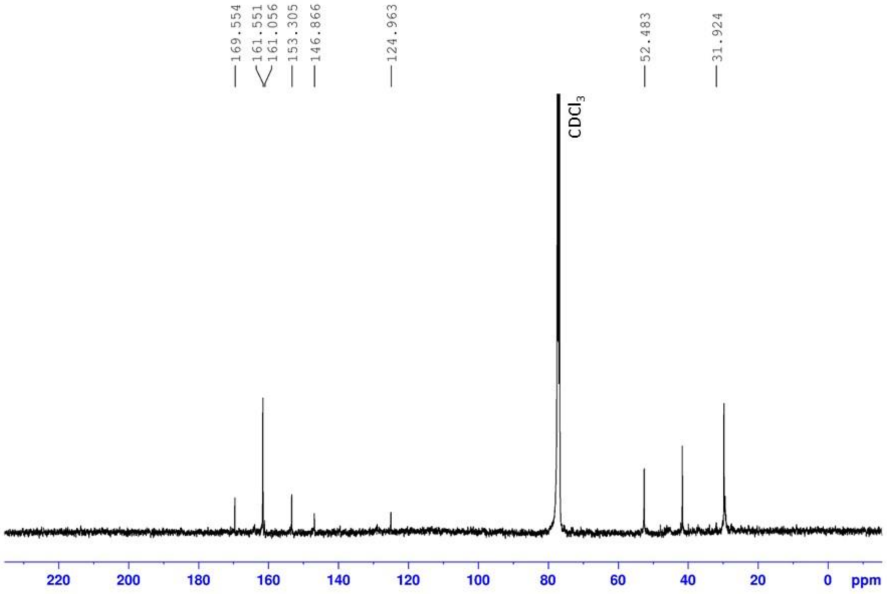

Compound **11b**

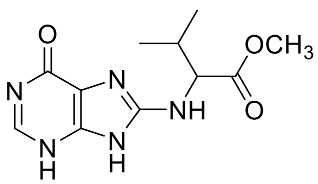

Supporting information

^{1}H-NMR

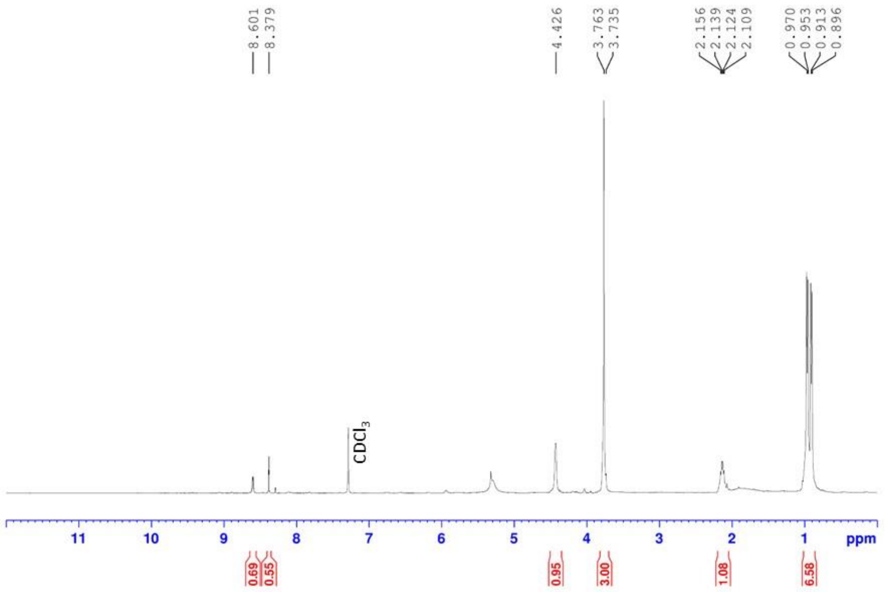

^{13}C-NMR

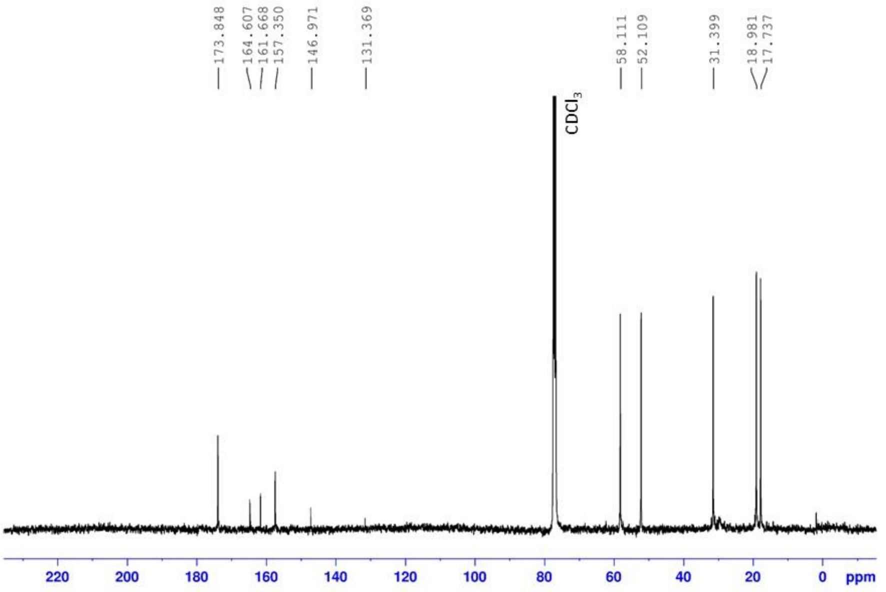

Supporting information

Compound **11c**

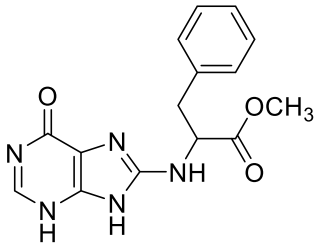

^{1}H-NMR

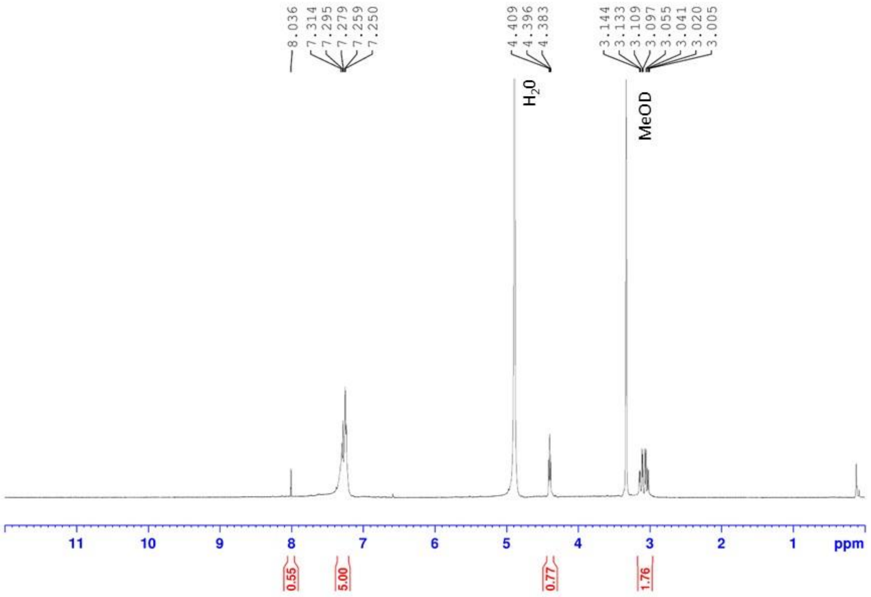

^{13}C-NMR

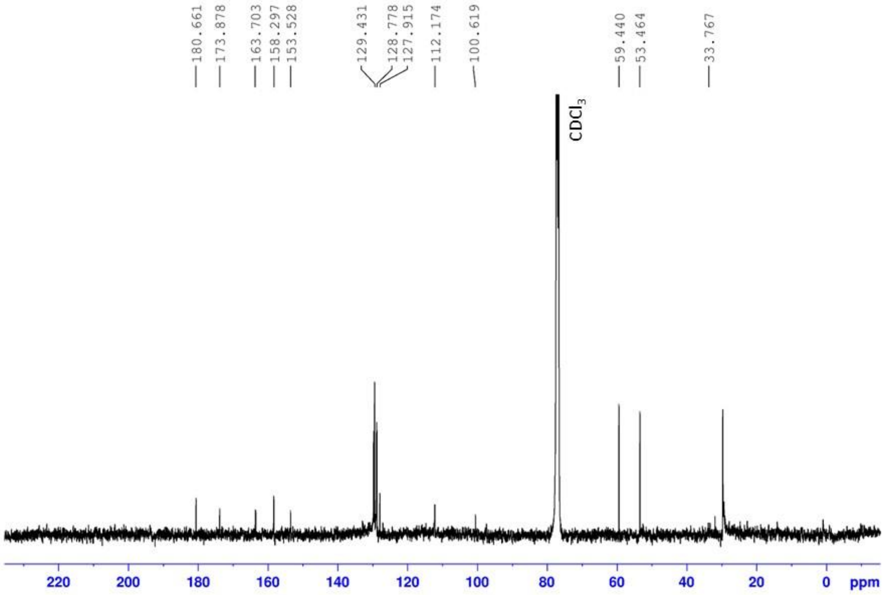

Supporting information

Compound **11d**

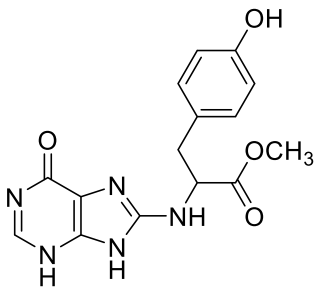

^{1}H-NMR

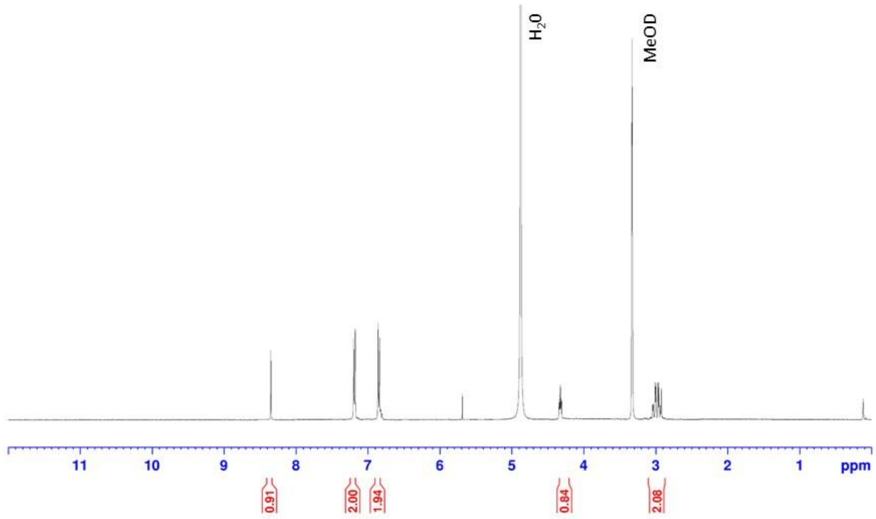

^{13}C-NMR

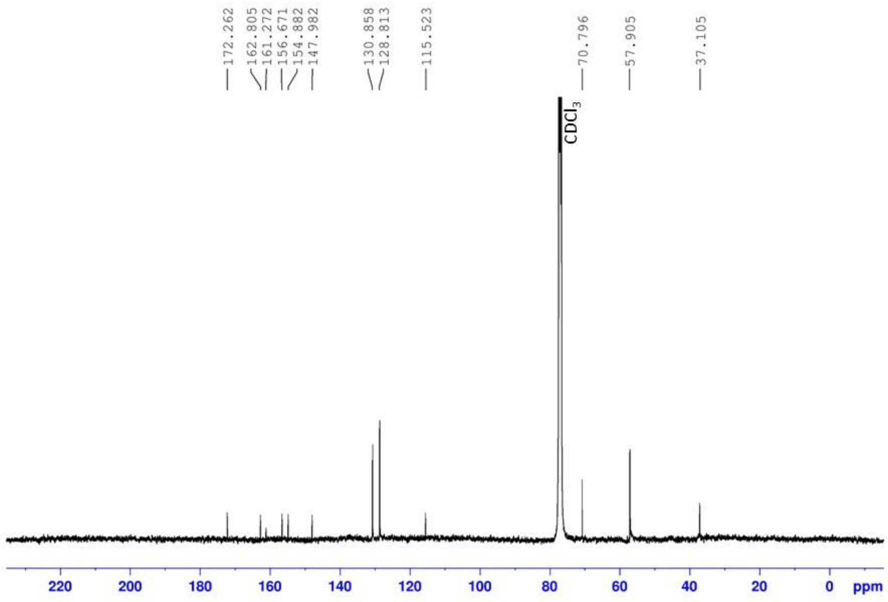

Supporting information

Compound **12a**

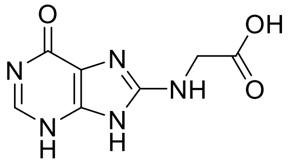

^{1}H-NMR

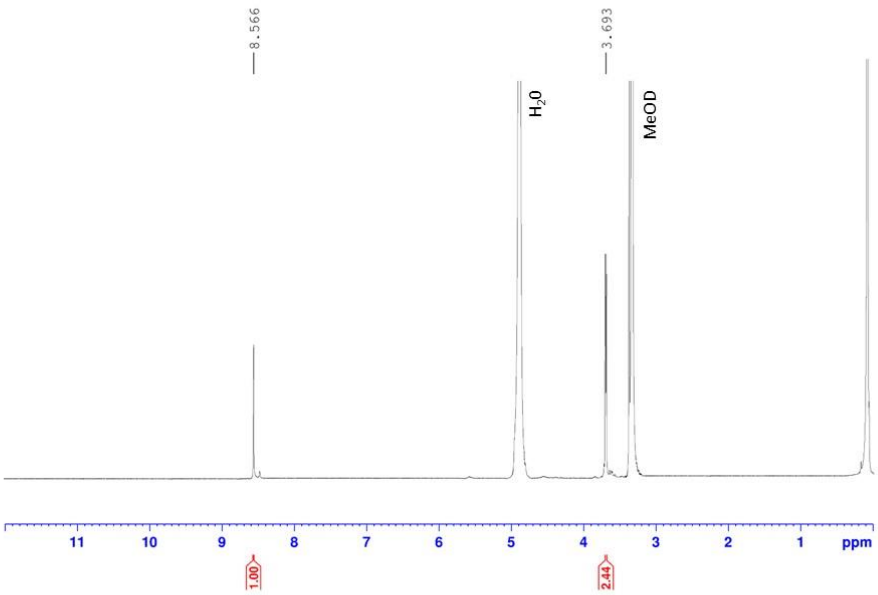

Compound **12b**

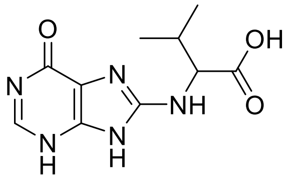

Supporting information

^{1}H-NMR

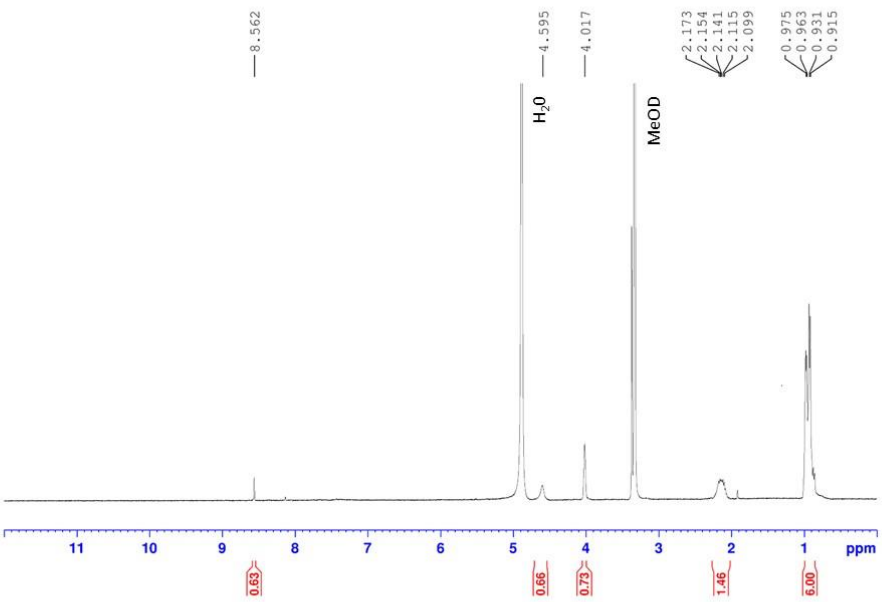

Compound **12c**

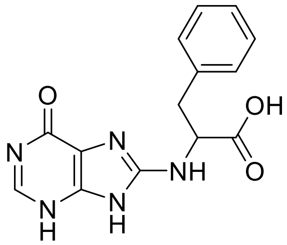

^{1}H-NMR

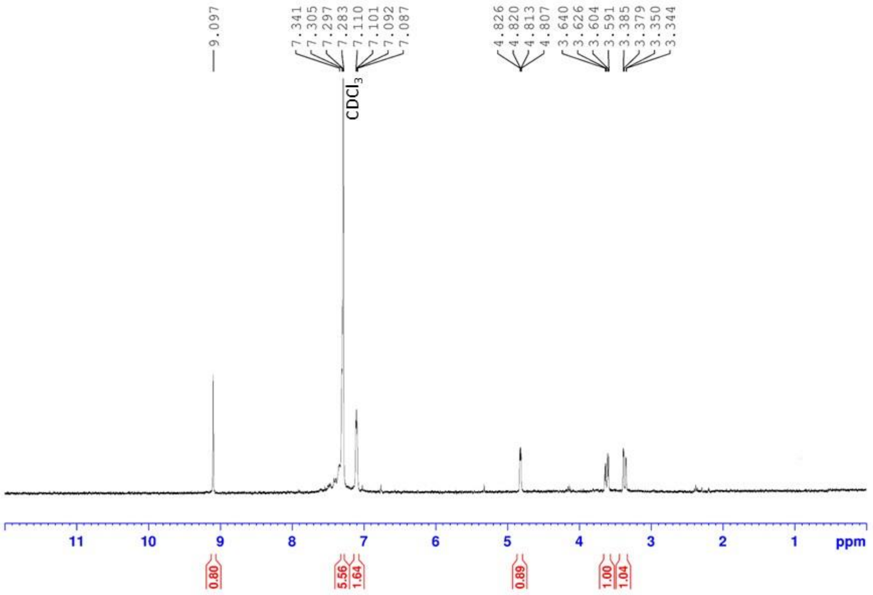

Supporting information

Compound **12d**

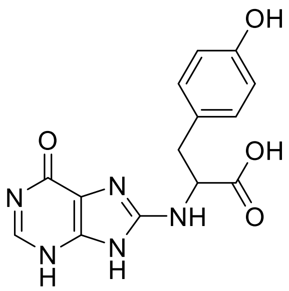

^{1}H-NMR

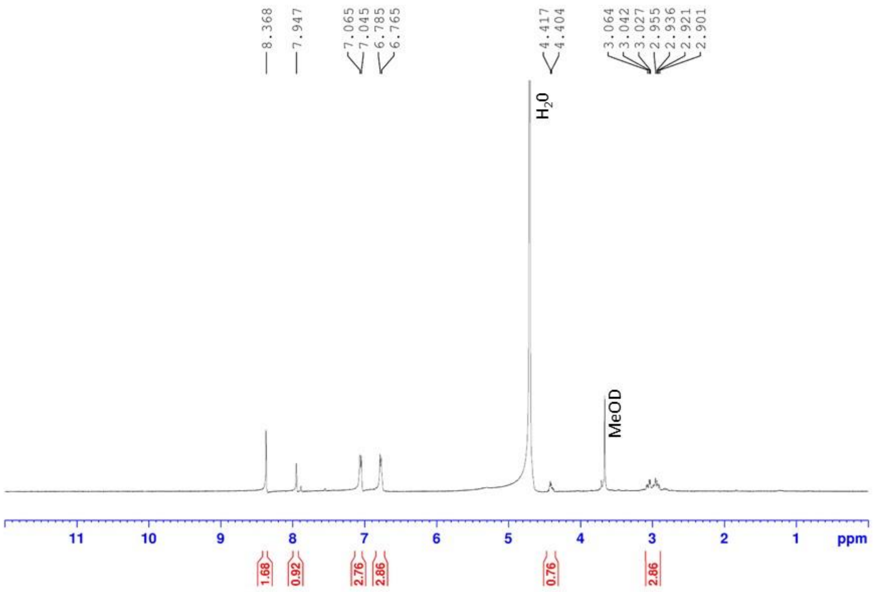

Supporting information

SI#2: Experimental details of two component reaction.

Reaction of 1 with 2

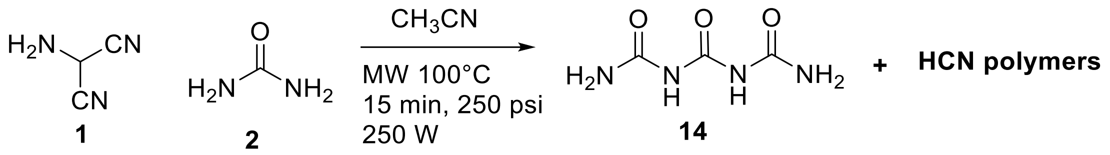

To a solution of Urea 2 (0.28 mmol) in CH_3CN (3 mL) was added triethylamine (0.24 mmol) and aminomalononitrile p-toluenesulfonate **1** (0.20 mmol). The mixture was stirred under microwave condition using the program in Table SI-T1:

Table SI-T1. Two component reactions of **1** with **2**

**Conditions**						**Conversion of 1**	**Yield of 14**	**Yield of HCN polymers**
N° of cycles	Temperature	Ramp Time	Hold Time	Pressure (psi)	Power (W)	40 %	20%	15%
3	100 °C	2 min	15 min	250	250			

At the end of the reaction, the solvent was removed under reduced pressure, the crude has been filtered and the soluble and the insoluble fractions analysed separately. The soluble fraction has been analysed by a Vanquish U-HPLC (Thermo scientific) associated to ISQ-EC MS(Thermo scientific) equipped with C18 Avantor ACE-5 (5 µm x 250 mm x 4.6 mm) performed the chromatographic separations using the following conditions: column temperature 25 °C, flow rate 0.2 ml/min, gradient elution with phase A (H_2O) and phase B (Methanol). The gradient employed was as follows:6 min 99% A; 15 min 96% A; 55 min 70% A, 64min 1%A, 68min 99% A. Products were detected by their absorbance at 254 and 205 nm. Product and yield of reaction have been assigned by comparison with commercially available standard compounds. The insoluble residue has been analysed by GC-MS after standard derivatization procedures with BSTFA and TMCS. In detail, in a round bottom flask *N*,*N*-bis-trimethylsilyl trifluoroacetamide (420 µL; Merck >99%) and a solution of pyridine (200 µL; Merck >99%) were added to 10 mg of crude of the reaction. The mixture was left under magnetic stirring at 90 °C for 4 h. Thereafter the solution was cooled down to 25 °C and 2.0 µl of the solution were used for the GC-MS analysis. Chromatographic conditions: CP8944 column (WCOT fused silica, film thickness 0.25 µm, stationary phase VF-5 ms, Øί 0.25 mm, length 30 m), injection temperature 280 °C, detector temperature 280 °C, gradient 100 °C x 2 min, then 10 °C/min for 60 min. GC-MS fragmentation spectra were recovered by using a triple quadrupole MS analyzer as full scan and single ion research modes. Detected mass-fragmentation values (m/z 149.4, 94.7 and 58.7) (Figure SI 2) are associated to the presence of isomeric HCN oligomers (Origins of life 1975, 6, 513-525).

Supporting information

Figure SI 1. Mass-fragmentation values of Triuret **14**

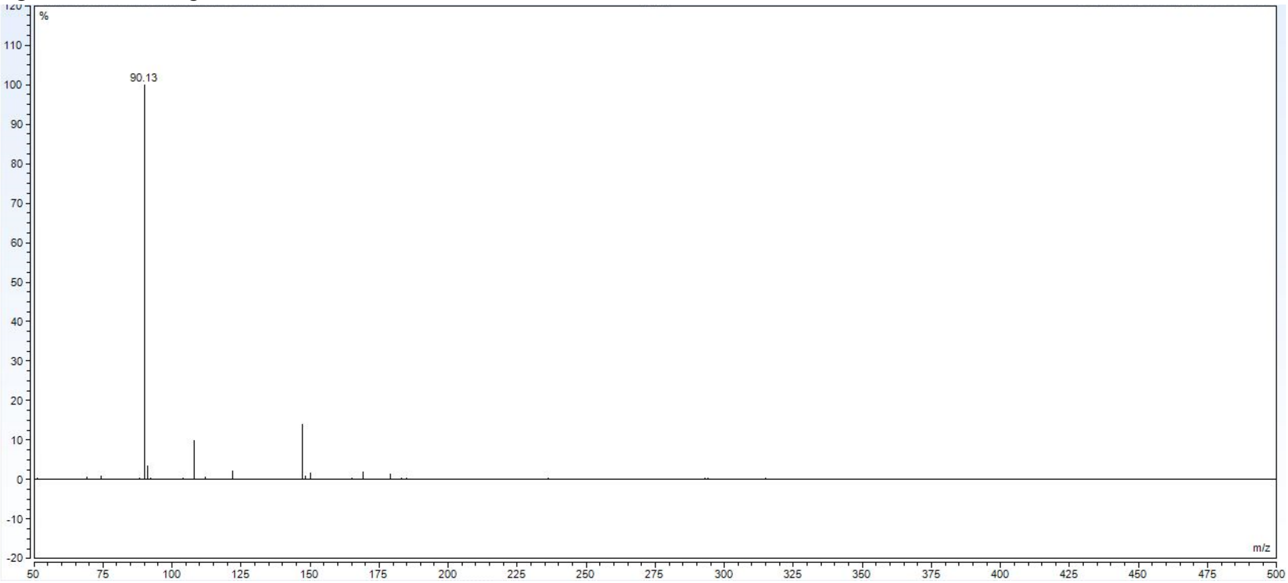

Figure SI 2. Mass-fragmentation values of peaks comprised in the retention time range between 23 min and 29 min

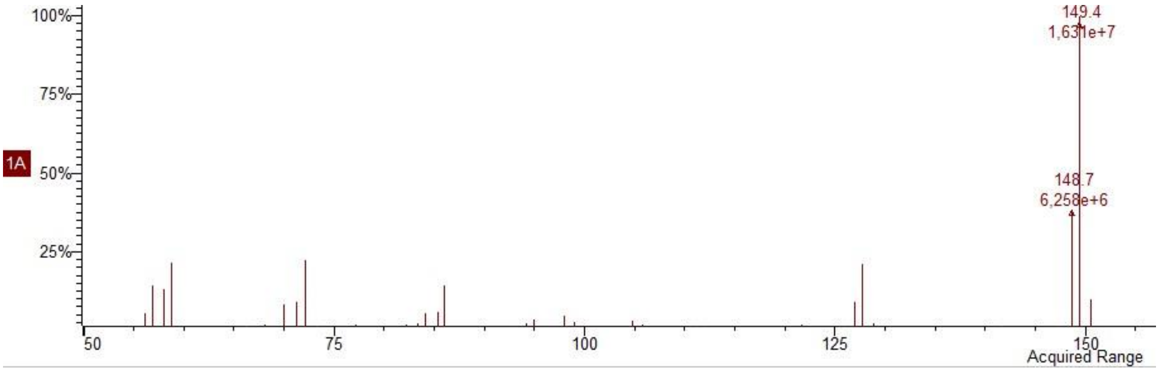

Supporting information

Reaction of 1 with 3a

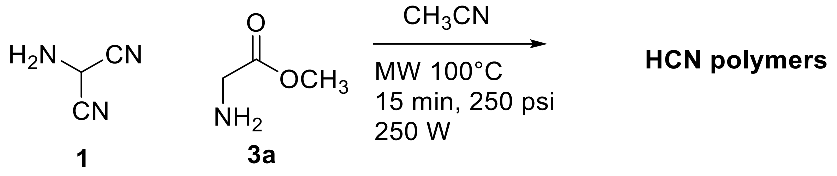

Scheme S1. Reaction of 1 with 3a

To a solution of **1** (0.20 mmol) in CH_3CN (3 mL) was added triethylamine (0.24 mmol) and a-amino-acid methyl ester **3a** (0.24 mmol). The mixture was stirred under microwave condition using the program in Table SI-T2:

Table SI-T2. Two component reactions of **1** with **3a**

Conditions						Conversion of 1	Yield of HCN polymers
N° of cycles	Temperature	Ramp Time	Hold Time	Pressure (psi)	Power (W)	30 %	25%
3	100 °C	2 min	15 min	250	250		

At the end of the reaction, the solvent was removed under reduced pressure and the insoluble residue has been analysed by GC-MS as described for reaction of **1** with **2**.

Figure SI 3. Mass-fragmentation values of peaks comprised in the retention time range between 23 min and 29 min

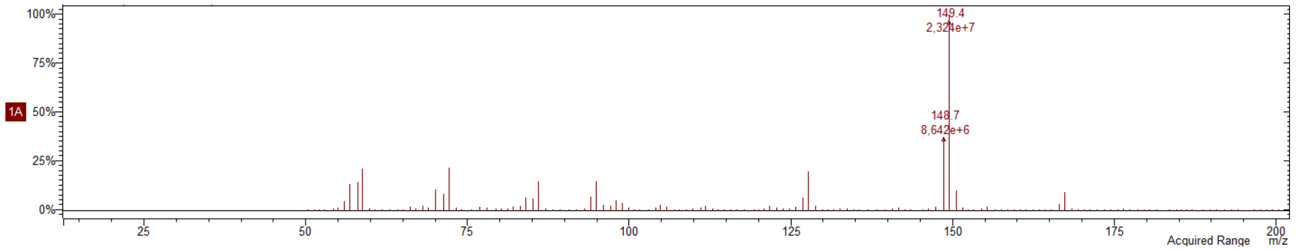

Supporting information

Reaction of 2 with 3a

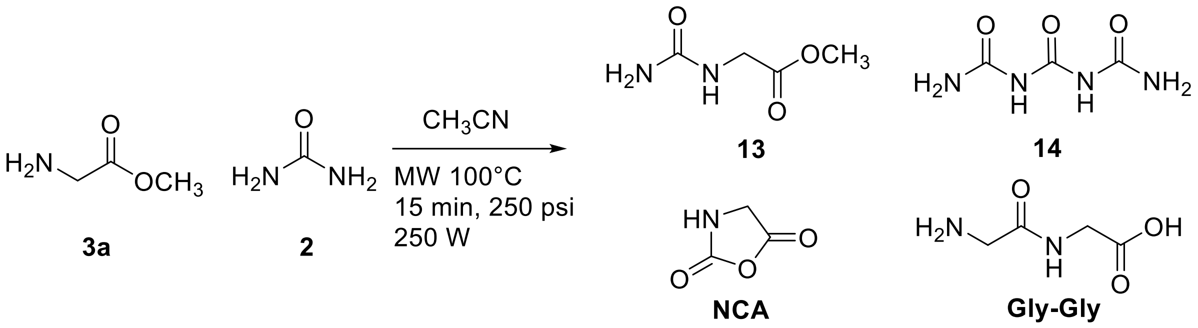

Scheme S2. Reaction of **2** with **3a**

To a solution of Urea **2** (0.28 mmol) in CH_3CN (3 mL) was added triethylamine (0.24 mmol) and α-amino-acid methyl ester **3a** (0.24 mmol). The mixture was stirred under microwave condition using the program in Table SI-T3:

Table SI-T3. Two component reactions of **2** with **3a**

**Conditions**						**Conversion of 3a**	**Yield of 13[a]**	**Yield of 14[a]**	**Yield of NCA[a]**	**Yield of Gly-Gly[a]**
N° of cycles	Temperature	Ramp Time	Hold Time	Pressure (psi)	Power (W)	63 %	13%	30%	14%	5%
3	100 °C	2 min	15 min	250	250					

[a] Yield is referred to the conversion of **3a.**

At the end of the reaction, the solvent was removed under reduced pressure and the crude has been analysed by HPLC-MS procedures Product and yield of reaction have been assigned by comparison with commercially available or ad-hoc synthesized standard compounds using co-injection method. A Vanquish U-HPLC (Thermo scientific) associated to ISQ-EC MS(Thermo scientific) equipped with C18 Avantor ACE-5 (5 µm x 250 mm x 4.6 mm) performed the chromatographic separations using the following conditions: column temperature 25 °C, flow rate 0.2 ml/min, gradient elution with phase A (H_2O) and phase B (Methanol). The gradient employed was as follows:6 min 99% A; 15 min 96% A; 55 min 70% A, 64min 1%A, 68min 99% A. Products were detected by their absorbance at 254 and 205 nm.

Supporting information

Figure SI 4. HPLC-MS profile of two component reaction of **3a** with **2**

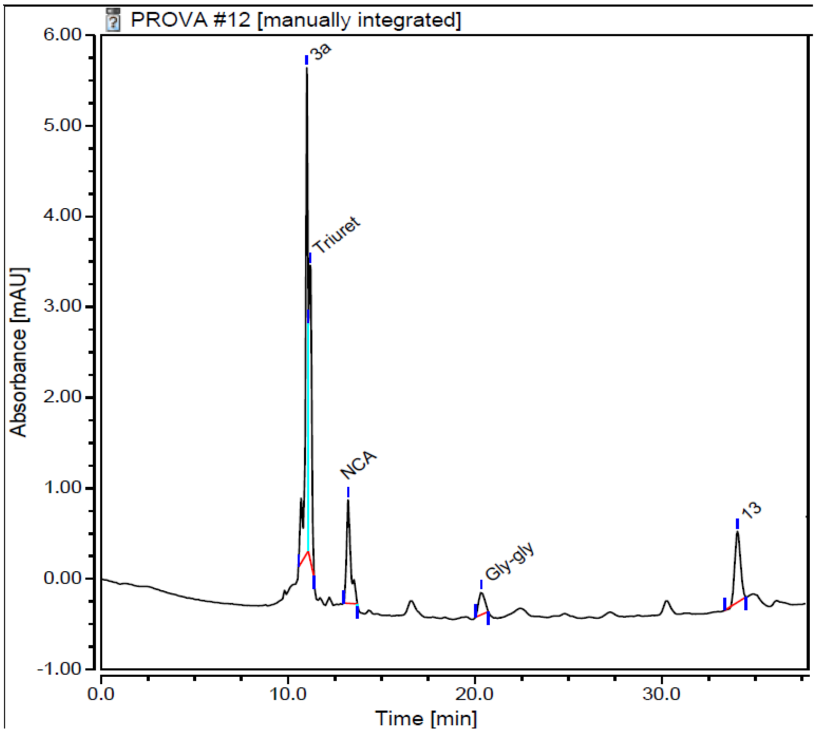

Figure SI 5. MS Fragmentation spectra of **3a**

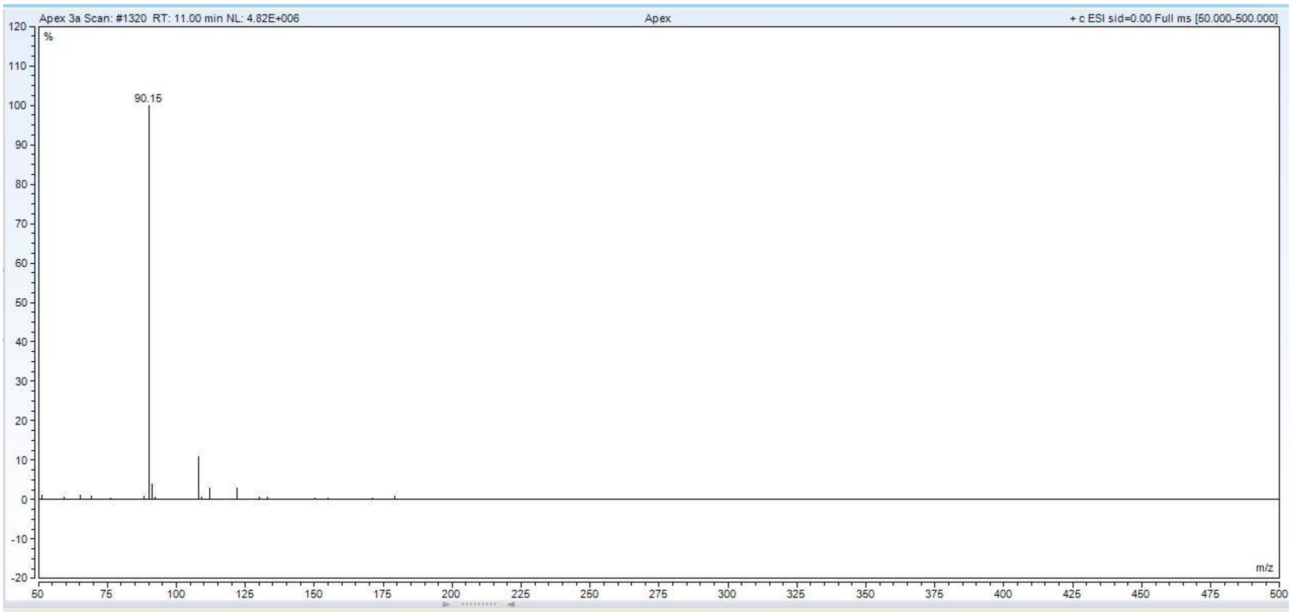

Supporting information

Figure SI 6. MS Fragmentation spectra of **Triuret 14**

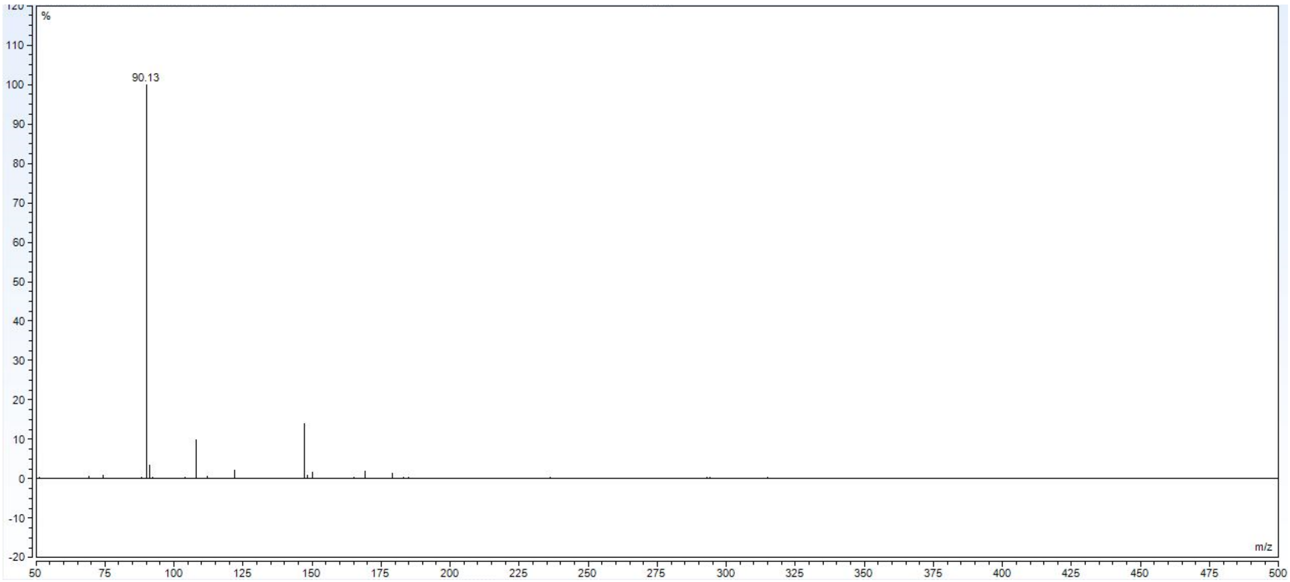

Figure SI 7. MS Fragmentation spectra of **NCA**

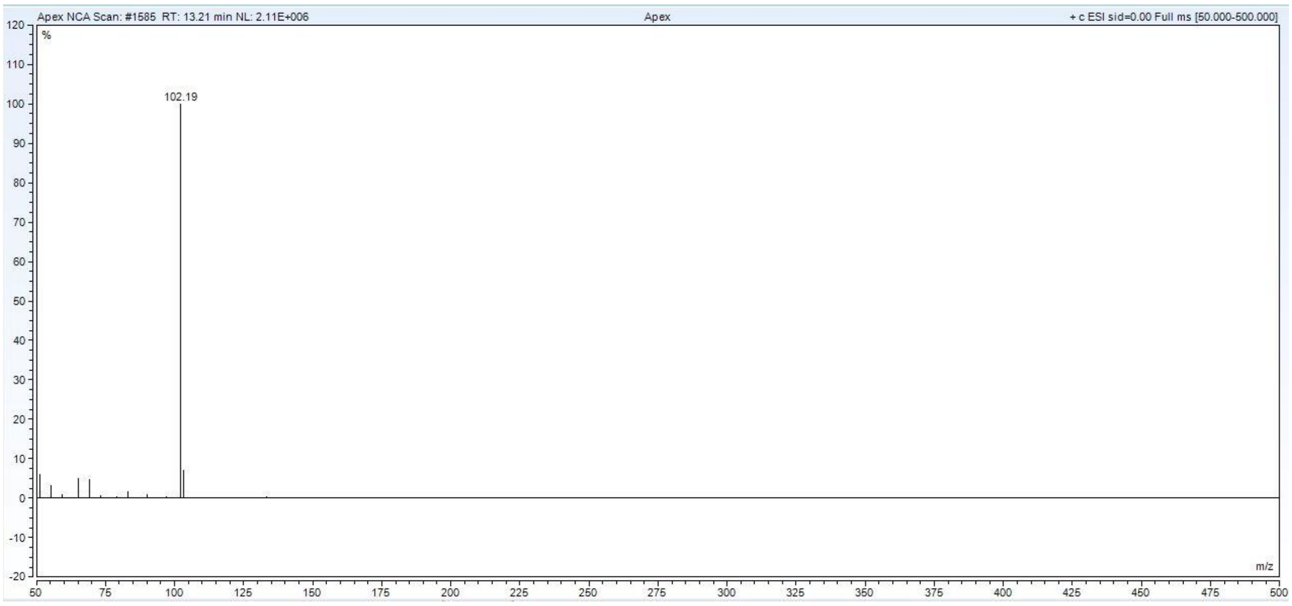

Supporting information

Figure SI 8. MS Fragmentation spectra of **Gly-Gly**

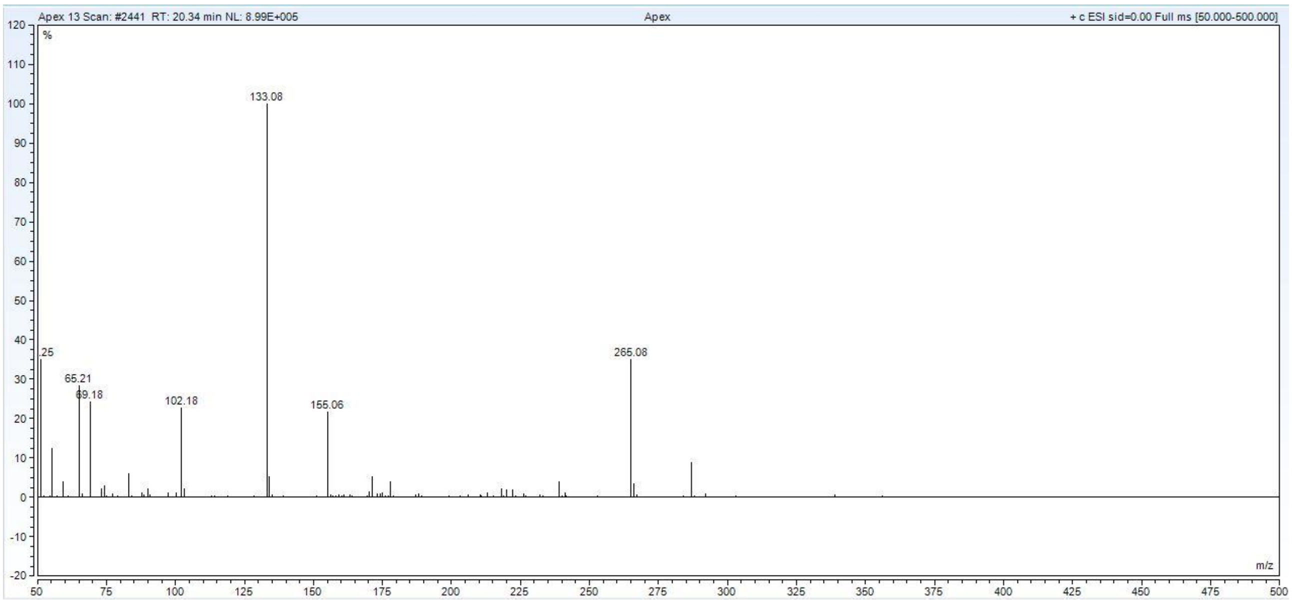

Figure SI 9. MS Fragmentation spectra of **13**

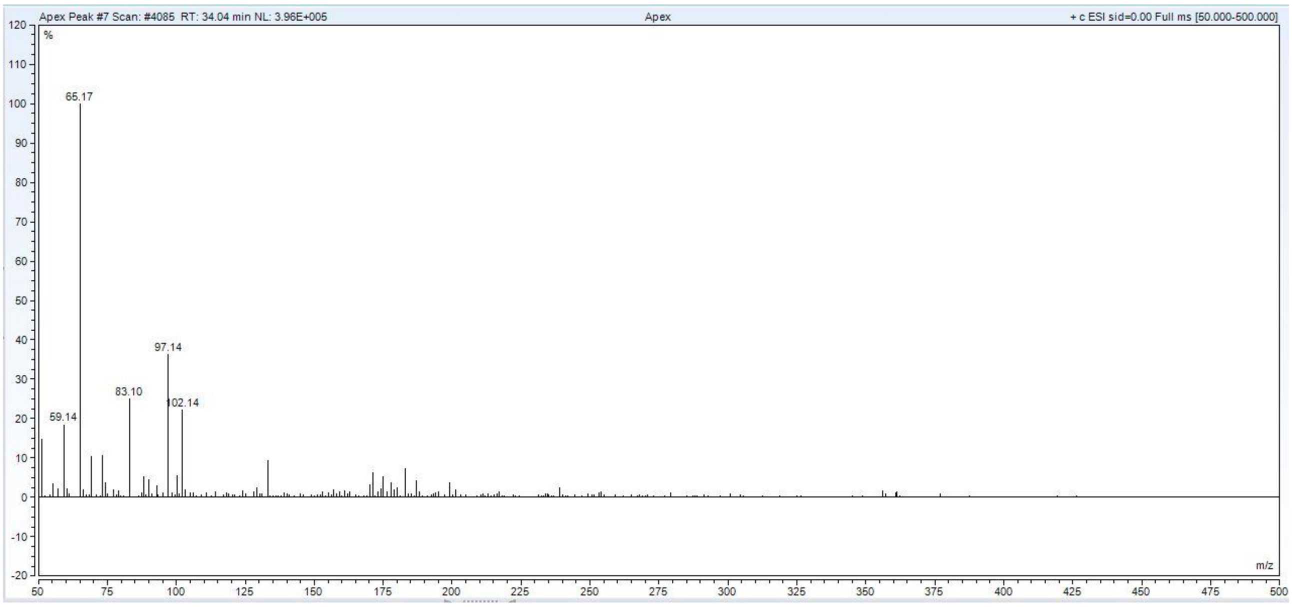

Supporting information

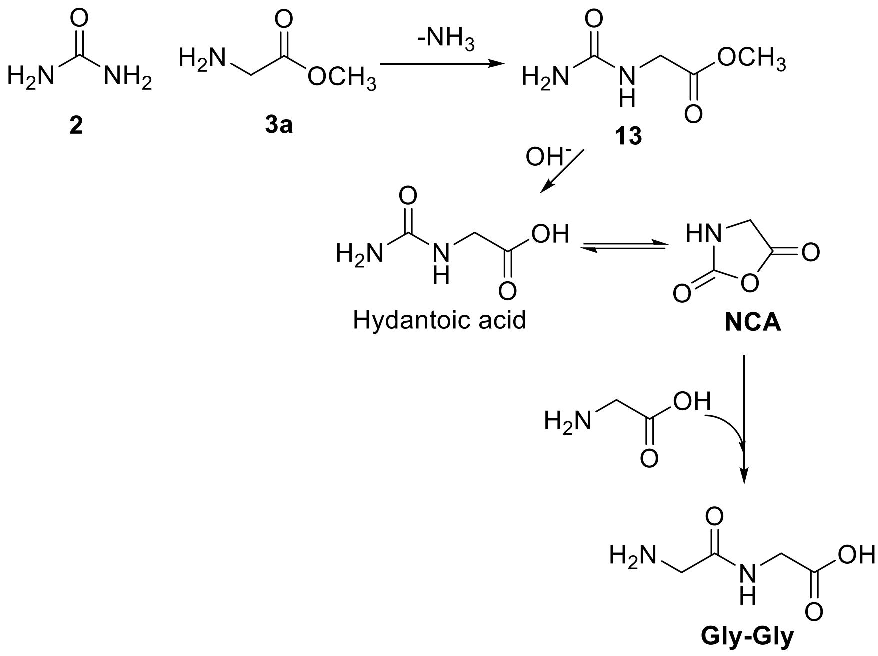

Scheme S3. Possible mechanism for the formation of NCA and Gly-Gly species.

3a reacted with **2** affording the corresponding N-carbamyl amino acid ester **13** . Then hydantoic acid (not isolated in the reaction crude), possibly produced due to the basic condition of the reaction from **13**, acts as a key role in its evolution into peptide **Gly-Gly t**hrough the formation of **NCA**. This reaction pathway is in accordance with data in literature concerning the mutual role of urea and aminoacids in the synthesis of peptides[45].

Supporting information

SI#3: Chromatographic profile of reactions under complete prebiotic conditions.

Products were detected by their absorbance at 254 and 205 nm. Assignment of chromatographic signals has been performed by comparison with standard compounds commercially available or ad-hoc synthesized. HPLC profile of reaction crude of **8a** under complete prebiotic conditions recorded at 254nm.

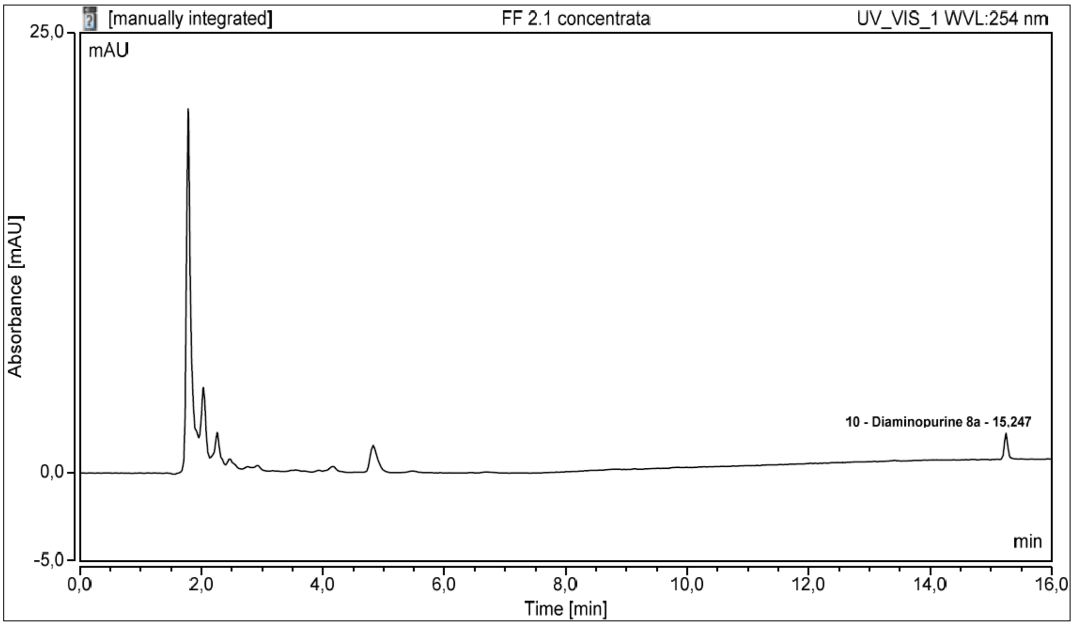

hydantoic acid rt: 4.9 min; diaminopurine **8a** rt: 15.2 min

HPLC profile of reaction crude of **8b** under complete prebiotic conditions.

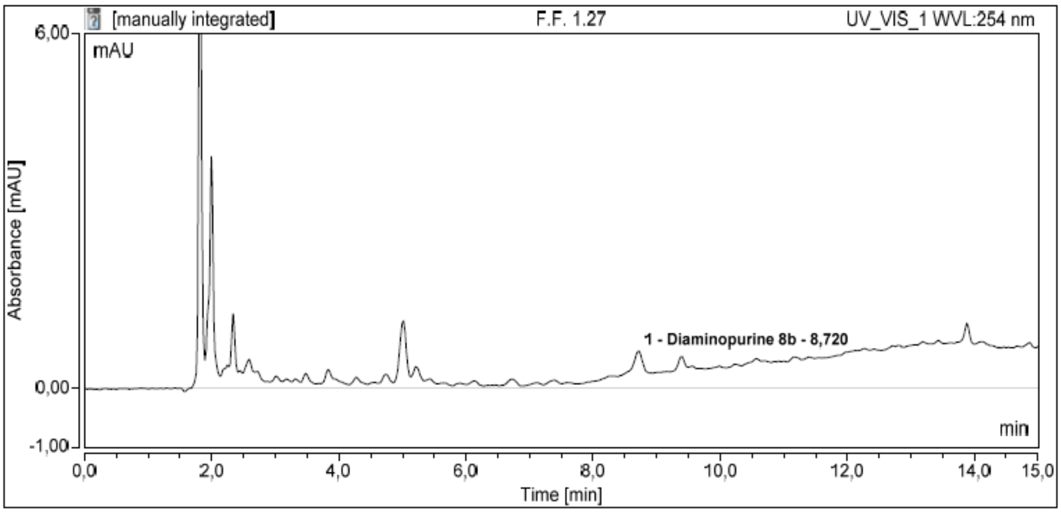

hydantoic acid rt: 4.9 min; diaminopurine **8b** rt: 8.7 min

Supporting information

HPLC profile of reaction crude of **8c** under complete prebiotic conditions.

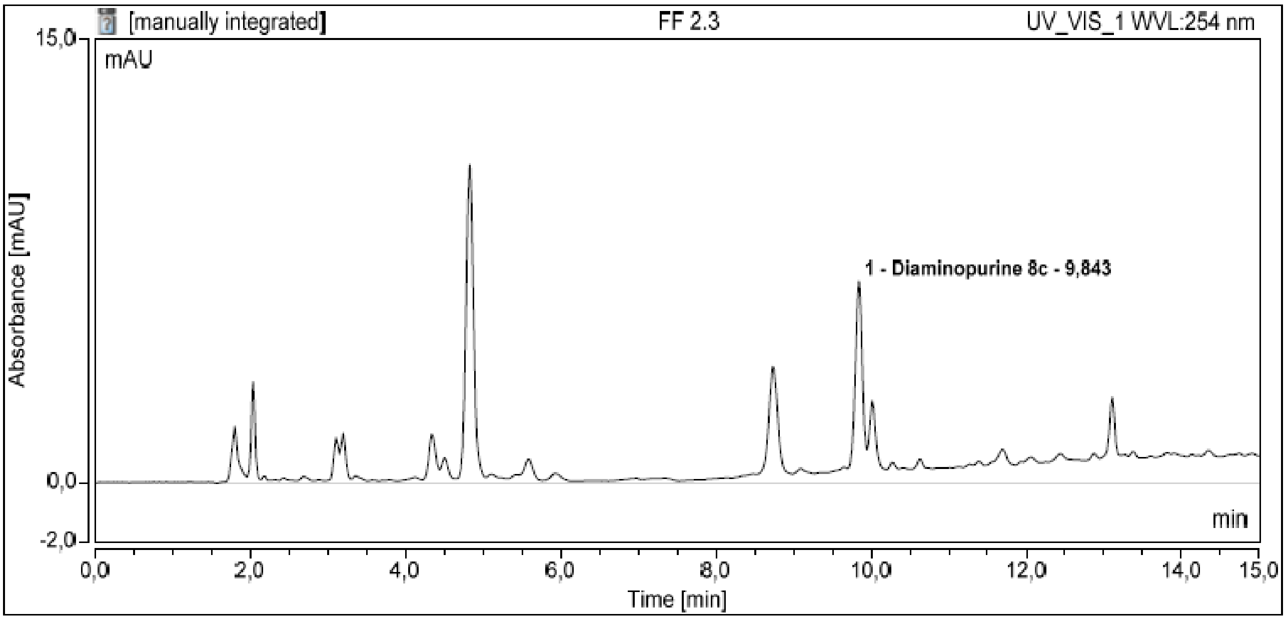

hydantoic acid rt: 4.9 min; diaminopurine **8c** rt: 9.8 min

HPLC profile of reaction crude of **8d** under complete prebiotic conditions.

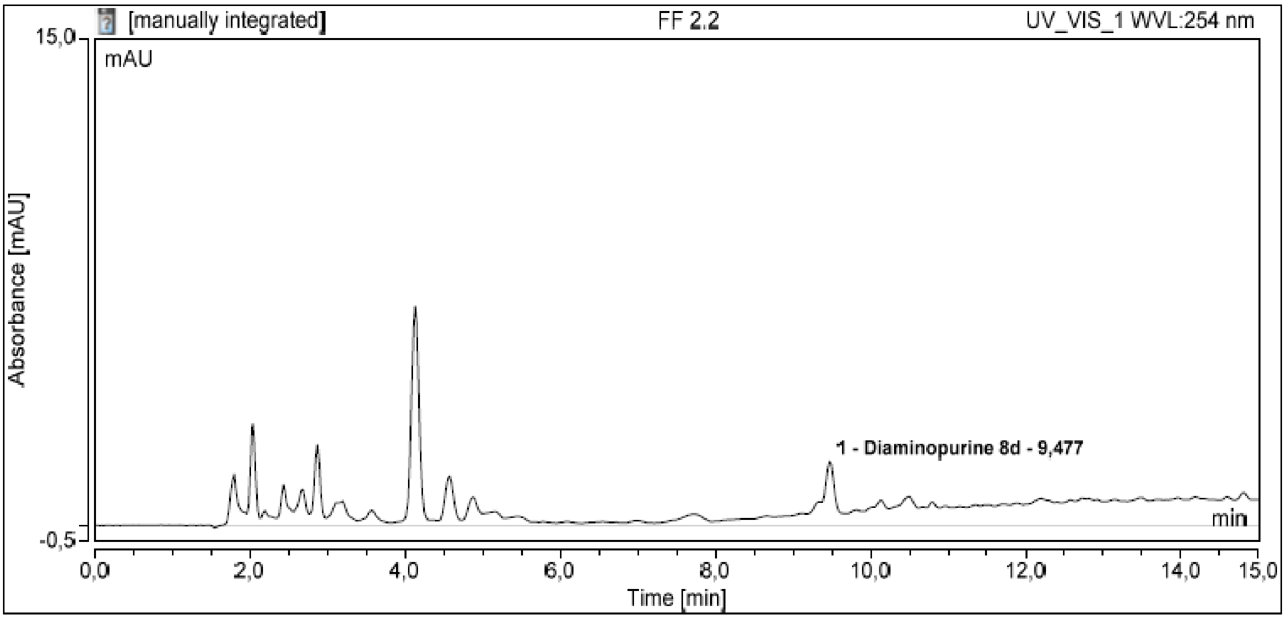

hydantoic acid rt: 4.9 min; diaminopurine **8d** rt: 9.4 min
